# Nuclear mechanosensing of the aortic endothelium in health and disease

**DOI:** 10.1242/dmm.050361

**Published:** 2023-11-01

**Authors:** Aarren J. Mannion, Lars Holmgren

**Affiliations:** Department of Oncology-Pathology, Karolinska Institute, Stockholm 171 64, Sweden

**Keywords:** Chromatin, Endothelium, Mechanosensing, Nuclear mechanics, Shear flow sensing, Vascular disease

## Abstract

The endothelium, the monolayer of endothelial cells that line blood vessels, is exposed to a number of mechanical forces, including frictional shear flow, pulsatile stretching and changes in stiffness influenced by extracellular matrix composition. These forces are sensed by mechanosensors that facilitate their transduction to drive appropriate adaptation of the endothelium to maintain vascular homeostasis. In the aorta, the unique architecture of the vessel gives rise to changes in the fluid dynamics, which, in turn, shape cellular morphology, nuclear architecture, chromatin dynamics and gene regulation. In this Review, we discuss recent work focusing on how differential mechanical forces exerted on endothelial cells are sensed and transduced to influence their form and function in giving rise to spatial variation to the endothelium of the aorta. We will also discuss recent developments in understanding how nuclear mechanosensing is implicated in diseases of the aorta.

## Teaser

**Summary:** Endothelial cells are exposed to mechanical forces such as shear stress exerted by blood flow. We explore the emerging role of the nucleus in how these forces are sensed and transduced.

## Introduction

The vessels of the circulatory system are lined by a continuous monolayer of endothelial cells (ECs), which are exposed to mechanical forces from circulating blood. Fluid shear stress acting on the endothelium of the aorta both maintains homeostasis and influences disease progression. Perturbation of mechanosensory pathways can impair endothelial function and eventually leads to highly prevalent diseases such as atherosclerosis (Hahn and Schwartz, 2009; Aitken et al., 2023). Mechanical forces are sensed through a variety of mechanisms and converted into biochemical signals in a process termed mechanotransduction, allowing ECs to adapt their form and function to the altered force environment and thereby maintain vascular homeostasis. In areas of unidirectional straight or laminar blood flow (LF), ECs elongate parallel to the direction of flow and phenotypically display atheroprotective properties (Davies, 1995). Conversely, areas of vessel bifurcation, curvature and branching create regions of oscillatory and disturbed flow (DF). In these regions, ECs display a more rounded morphology and a proinflammatory profile and, subsequently, these areas are prone to plaque development and atherosclerosis (Davies, 1995).

In this Review, we discuss recent developments in the field of mechanobiology of the endothelium of the aorta. Although stretching and extracellular matrix (ECM) stiffness profoundly influence aortic EC biology, the relatively low piconewton force exerted by shear stress dominates EC phenotypes, and thus we focus on recent work describing how the aortic endothelium senses differential flow profiles. Moreover, we discuss how fluid shear stress is sensed by the cell and transduced to the nucleus either directly or indirectly, and how this informs intranuclear dynamics to shape chromatin conformation and, ultimately, transcriptional responses. The flow-dependent transcriptional response of ECs is well documented, so our focus will be on recently reported mechanosensors and mechanotransducers, and on the role of the nucleus and chromatin arrangements in shaping these transcriptional responses. Finally, we evaluate evidence of dysfunctional endothelial mechanosensing and its relevance for pathologies of the aorta.

## Shear stress profile of the aorta

**Fig. 1.**
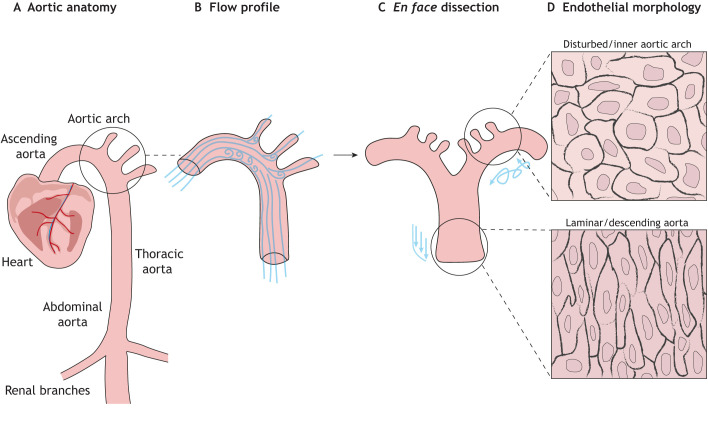
**Aortic architecture, flow profile and cellular morphology of the endothelium.** (A) The aorta is the main conduit of oxygenated blood to the rest of the body. (B) The architecture of the aortic arch produces differential shear flow profiles, with disturbed flow at the inner arch and branch points to other vessels, and laminar flow in the ascending and descending regions of the aorta. (C) *En face* preparation, using the open book or Häutchen method (Obaze and Wright, 1968), allows examination of the endothelial monolayer. (D) Imaging of the dissected aorta has revealed that endothelial morphology concurs with differing shear flow profiles exerted on different regions of the aortic vessel wall.

Blood ejected from the left ventricle during the cardiac cycle travels through the ascending aorta, the aortic arch and finally down the descending aorta (Fig. 1A). In the aortic arch, the shear stress profile of the blood is dictated by the hairpin-like loop of the aorta creating regions of differential frictional stress acting on the immediate aortic wall. This anatomical structure gives rise to DF and LF profiles, with a region of DF in the aortic arch that transitions to LF as blood moves down the descending aorta. Time-resolved three-dimensional magnetic resonance imaging of blood moving through the aorta has revealed a detailed picture of the complex profile of fluid dynamics (Oechtering et al., 2020). A turbulent and disturbed flow profile is present within the inner aortic arch, whereas at the outer regions of the arch, the shear stress profile is more uniform. As the blood continues around the bend of the aortic arch, a unidirectional, laminar shear stress profile is maintained down into the descending portion. The branch points to the carotid, subclavian and brachiocephalic arteries, positioned at the outer aortic arch, give rise to smaller regions of DF as the LF encounters the perpendicular vascular wall (Fig. 1B). The velocity of blood is also affected by the shape of the aorta, with the greatest velocity observed in the ascending and descending aorta, whereas the DF region in the inner aortic arch exhibits reduced velocity (Oechtering et al., 2020).

Flow patterning forms during development and is maintained throughout life. DF in the aorta occurs at postnatal day (P) 5 when the ductus arteriosus closes due to shifting of ventricular pressure induced by inflation of the lungs at birth. This was recently highlighted by Hernandez et al. (2022), who showed that this onset of flow patterning can dictate EC morphology of the aortic arch. In humans, the condition patent ductus arteriosus, in which normal closure of the ductus arteriosus at birth does not occur, results in significantly increased mortality and vascular diseases among patients (Schneider and Moore, 2006). However, although it is known that endothelial and smooth muscle cell remodelling underlies this adaptation (Salvador et al., 2022) and impacts flow dynamics in preterm infants (Broadhouse et al., 2015), a direct link between patent ductus arteriosus and the onset of DF and impaired EC mechanosensing in humans is unknown.

## Endothelial integration of shear stress

Mechanical forces profoundly influence cellular and nuclear morphology. The hypothesis that mechanical force can influence nuclear shape was posited by Champy and Carleton in the 1920s (Champy and Carleton, 1921). Following this hypothesis, morphological adaptation of ECs to fluid shear stress was reported in the 1950s, and ECs were described to align in the direction of blood flow (Altschul, 1954). Around twenty years later, in-depth studies of canine aorta using silver nitrate staining and whole-mount *en face* dissections (Fig. 1C) showed altered nuclear orientation in different regions (Flaherty et al., 1972), and *en face* preparations of rabbit aorta confirmed that regions exposed to LF exhibit elongated nuclei parallel to the direction of flow and more rounded nuclei in regions of disturbed non-uniform flow in the inner aortic arch (Fig. 1D) (Silkworth et al., 1975). The impact of flow on EC morphology was further demonstrated by the partial removal of canine thoracic aorta and its surgical reinsertion at 90° to the direction of flow for 10 days (Flaherty et al., 1972). This reorientated the nuclei and cell bodies parallel to the direction of LF, showing that EC morphology was influenced by shear stress.

The development of more sophisticated biomechanical approaches to study ECs under shear stress led to a great number of studies elucidating endothelial responses to fluid shear stress. These confirmed that shear stress influences EC shape (Dewey et al., 1981) and proliferation (Davies et al., 1986) and that it maintains both pro- and anti-inflammatory signalling pertinent to vascular homeostasis (Nagel et al., 1994; Ohtsuka et al., 1993). Advancements in understanding the transcriptional changes in ECs exposed to differing patterns and magnitudes of shear stress led not only to seminal findings regarding shear stress-induced gene expression (discussed below), but also to further questions regarding the cellular apparatus responsible for the primary sensing of shear stress to transduce mechanical signals into biochemical responses.

## Endothelial mechanosensory mechanisms

### Junctions

A variety of mechanisms sense shear stress, the most well-known being the VE-cadherin (or CDH5), PECAM1 and VEGFR2 (or KDR) mechanosensory complex within endothelial cell-cell junctions (Tzima et al., 2005). This complex is activated when shear stress induces PECAM1-dependent activation of the non-receptor tyrosine kinase Src, which then facilitates ligand-independent activation of VEGFR2. VEGFR2 phosphorylation then triggers downstream activation of the p85 subunit of the phosphoinositide 3-kinase family, AKT kinases and integrins (Tzima et al., 2005). Subsequent work showed that tension across PECAM1 increases upon exposure of ECs to flow, whereas the tension experienced by VE-cadherin decreases (Conway et al., 2013). Flow-mediated PECAM1 tension was due to an association between PECAM1 and vimentin, which, when depleted, also led to defects in EC flow alignment (Conway et al., 2013).

The junctional mechanosensory complex was further expanded to include VEGFR3 (or FLT4) through its association with the transmembrane domain of VE-cadherin. This VE-cadherin domain is critical for binding both VEGFR2 and VEGFR3, triggering downstream signal transduction and the alignment of ECs to shear stress. Activation and phosphorylation of VEGFR3 in response to shear stress occurs in both lymphatic and vascular ECs. Additionally, VEGFR3 was found to be highly expressed in the ECs of the inner aortic arch and its deletion led to reduced inflammatory markers (Coon et al., 2015). Further work from the Schwartz laboratory showed that VEGFR3 acts as a fluid shear stress sensor that regulates inward vascular remodelling, which is crucial in maintaining vascular homeostasis and regulating shear stress (Baeyens et al., 2015).

The transmembrane receptor plexin D1 plays an important role in directly sensing fluid shear stress, as conformational change of this receptor is required for downstream mechanotransduction (Mehta et al., 2020). Binding of plexin D1 to neuropilin 1 and VEGFR2 occurs under shear stress and triggers downstream flow-responsive signalling, highlighting plexin D1 as an upstream mechanosensor in the junctional mechanosensory complex (Mehta et al., 2020). Additionally, deletion of plexin D1 in aortic ECs resulted in reduced plaque development (Mehta et al., 2020). Plexin D1 also responds to both LF and DF through the regulation of the Krüppel-like family of transcription factors (KLFs) and the proinflammatory genes *CCL2* and *VCAM1*, suggesting its differential abilities in sensing flow profiles (Mehta et al., 2020).

Recently, our group identified another junctional shear stress-sensing molecule, angiomotin-like protein 2 (AmotL2), that binds VE-cadherin and p120 catenin (CTNND1) in ECs (Zhang et al., 2023). The onset of LF triggered the association of AmotL2, VE-cadherin and p120 catenin with the actin cytoskeleton, forming a contiguous pathway from cell-cell junctions to the nuclear membrane. Additionally, *in vivo* deletion of endothelial AmotL2 led to impaired EC alignment and nuclear morphology (Zhang et al., 2023) and, as shown in recently preprinted work, impacts on chromatin accessibility (Mannion et al., 2023 preprint). In a strikingly analogous role to AmotL2, neuropilin 1 was also shown to bind VE-cadherin in a flow-dependent manner, promote flow-induced association of p120 catenin and VE-cadherin, and regulate downstream anti-inflammatory signalling (Bosseboeuf et al., 2023).

### Focal adhesions and integrins

**Fig. 2.**
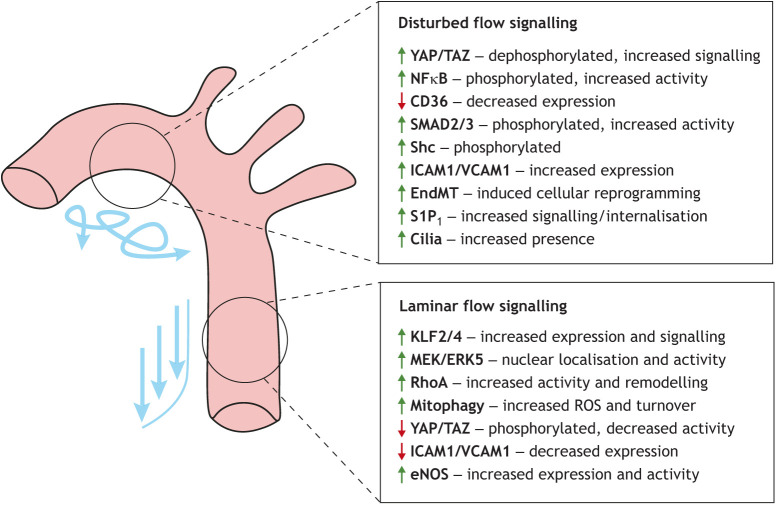
**Spatial signalling of the aortic endothelium.** Disturbed and laminar flow of the inner aortic arch and descending portion of the aorta, respectively, trigger regional signalling concomitant with the shear profile. The predominantly atherogenic, pro-inflammatory signalling is found in the aortic inner arch that is subjected to disturbed flow, whereas the atheroprotective, anti-inflammatory signalling is exhibited in the descending aorta, where endothelial cells experience laminar flow conditions. Green upward arrows indicate increased signalling activity, whereas red downward arrows indicate reduced activity. EndMT, endothelial-mesenchymal transition; ROS, reactive oxygen species.

Shear stress activates integrins, increases the activity and remodelling of focal adhesions and ECM binding, and regulates Rho-dependent cytoskeletal dynamics (Tzima et al., 2002; Davies et al., 1994, 1993) (Fig. 2). The junctional mechanosensory complex described above was discovered by probing downstream activation of integrins using an antibody against integrin α_V_β_3_ (Tzima et al., 2001), which highlighted the crosstalk between cell-cell junctions and basal cell-ECM mechanosensing. Src and the signal transducer Shc protein family, known to carry sequence homology to Src, were implicated in flow-responsive signalling, as regions of the aorta exposed to DF exhibited increased levels of phosphorylated (phospho-)Shc (Liu et al., 2008) (Fig. 2). In the same study, increased levels of phospho-Shc were subsequently observed for *in vitro* oscillating flow compared to the basal levels for LF, which were comparable to those for static conditions. This activation of Shc depended on VEGFR2 and VE-cadherin association and subsequent downstream flow-induced Src activity. Additionally, flow-activated phospho-Shc was found to associate with integrins in a VE-cadherin-dependent manner, further highlighting a mechano-induced crosstalk between integrins and cell-cell junctions (Liu et al., 2008). More recently, Mehta and colleagues demonstrated that exposure to shear stress enhanced the association between Shc and Alk5 (also known as TGFBR1) (Mehta et al., 2023). In this study, DF-mediated phosphorylation of Shc and activation of downstream SMAD signalling was found to be reliant on Alk5 expression and led to the endothelial-mesenchymal transition (EndMT) (Fig. 2) and to the development of atherosclerosis.

### G protein-coupled receptors

G protein-coupled receptors (GPCRs) are a diverse family of multipass transmembrane receptors and are well-characterised mechanosensors (Chachisvilis et al., 2006). To date, a number of GPCRs have been implicated in endothelial mechanosensing of shear stress (see Aitken et al., 2023 and Hu et al., 2022 for reviews on GPCR endothelial mechanosensing). Despite this, the precise implications of many of these mechanosensory GPCRs remain unknown in an aortic endothelial setting. The recently discovered GPR68 has been implicated in flow sensing and remodelling of mesenteric arteries (Xu et al., 2018a); however, its role in EC mechanosensing of the aorta remains to be explored. Key studies highlighting the role of GPCRs in the aortic endothelium include those on the sphingosine 1 phosphate receptor 1 (S1P_1_ or S1PR1), which is localised to EC junctions under LF conditions in the aorta and exhibits cytoplasmic endosomal localisation in ECs of the DF-exposed inner aortic arch (Jung et al., 2012) (Fig. 2). Furthermore, deletion of endothelial *S1pr1* led to impaired cell and nuclear shape, and alignment to LF in the descending aorta, accompanied by impaired anti-inflammatory phospho-eNOS (NOS3) activity (Jung et al., 2012). Central to GPCR signalling, G protein subunits such as the G_q_/G_11_ family are required for sensing of both DF and LF, endothelial deficiency of which reduced *Vcam1* expression in the aorta in regions of DF (Albarrán-Juárez et al., 2018) (Fig. 2). Another subunit, Gα_13_, regulates integrin activity under LF and subsequently YAP/TAZ nuclear localisation (Wang et al., 2016b). More recently, endothelial expression of the G_s_-coupled receptor calcitonin receptor-like receptor (CALCRL) was found to regulate endothelial eNOS expression under LF conditions (Iring et al., 2019) and NFκB responses to DF (Nakayama et al., 2020) (Fig. 2).

### Ion channels

Ion channels are exquisitely sensitive to mechanical force, altering their conformation upon mechanical stimulation (Martinac, 2004). The best-characterised ion channel in endothelial shear stress responses is Piezo1, which is required for shear stress-induced endothelial alignment, influx of Ca^2+^ and remodelling of focal adhesions in response to flow (Li et al., 2014). Both LF and DF activate a signalling pathway via Piezo1 and the GPCRs G_q_/G_11_ and P2Y_2_ (P2RY2) (Albarrán-Juárez et al., 2018). Under DF, this pathway activates the junctional mechanosensory complex and integrins, triggering NFκB activity and inflammation, which culminates in atherosclerosis. Furthermore, work in zebrafish embryos demonstrated that Piezo1 responds to mechanical forces during heart valve development, where it stimulates endothelial expression of the transcription factor KLF2 (Duchemin et al., 2019). Piezo1 also plays a role in the flow-induced mitochondrial pathway leading to ERK activation and upregulation of KLF2 (Coon et al., 2022). Interestingly, Piezo1 associates with PECAM1 and VE-cadherin at the EC cell-cell junction, where it maintains Ca^2+^ influx and actin cytoskeleton dynamics under flow conditions (Chuntharpursat-Bon et al., 2023).

### The glycocalyx

The glycocalyx, a layer of sialic acid and glycosaminoglycans, including hyaluronic acid (HA) and heparan, is tethered to the apical membrane by the HA receptor CD44 (Aruffo et al., 1990) and mediates shear flow sensing by ECs (Pahakis et al., 2007; Mochizuki et al., 2003). Previous work has outlined that the glycocalyx becomes degraded under DF conditions, is eroded in the inner aortic arch and regulates caveolin-1 (CAV1) expression and eNOS signalling (Harding et al., 2018). Additionally, recent evidence suggests that enzymatic cleavage of heparan sulphate promotes flow-induced angiopoietin-2 expression via AMPK/FoxO1 signalling in a KLF2-independent manner (Richter et al., 2022). A pathway linking the glycocalyx, apical CD44 expression and the intracellular spectrin cytoskeletal network of short, stabilised actin filaments was found to regulate shear stress-induced alignment of ECs (Mylvaganam et al., 2022). This alignment is junction independent, as sub-confluent ECs aligned under flow conditions *in vitro*, a phenomenon that requires HA, the glycocalyx, CD44 and the spectrin network. The same study confirmed the importance of spectrin network integrity for maintaining aortic EC alignment *in vivo*. Furthermore, the authors showed that shear stress altered plasma membrane tension, which subsequently triggered caveolae-localised Piezo1 activity and Ca^2+^ influx, which was required for downstream mechanosignalling and EC alignment (Mylvaganam et al., 2022). This finding opens further exciting questions about the glycocalyx and the HA-CD44-spectrin mechanosensory module. Can the HA-CD44-spectrin pathway differentially sense LF and DF? Is nuclear morphology affected by this mechanosensory module and is flow-regulated transcriptional response informed by the glycocalyx? It will be interesting to see what future studies of the mechanosensory glycocalyx show.

### Cilia

The role of cilia as endothelial sensors of fluid shear stress is less well studied despite evidence that cilia indeed act as primary sensors of flow (Nauli et al., 2008; AbouAlaiwi et al., 2009). Shear stress deforms cilia, triggering the downstream calcium signalling required for endothelial responses to flow (Goetz et al., 2014). Furthermore, ECs express cilia with a specialised structure that allows flexibility in response to flow (Goetz et al., 2014). In the mammalian aorta, primary cilia are present in regions of low DF (Van der Heiden et al., 2008) (Fig. 2) and regulate atherosclerosis (Dinsmore and Reiter, 2016). Additionally, recent work suggested that deciliation of ECs under high-flow conditions could even be a potential biomarker of endothelial damage (Gupta et al., 2022). The role of cilia in the context of EC flow sensing is extensively reviewed in Luu et al. (2018).

## Spatial signalling of the aortic endothelium

The architecture, site-specific flow dynamics and mechanosensory downstream signalling trigger the appropriate transcriptional response of the endothelium (summarised in Fig. 2). The conformational flexibility and structural adaptability of mechanosensors are thought to be the defining features of their ability to sense both LF and DF. For example, the open and closed ligand-bound conformation of plexin D1 plays a pivotal role in its mechanosensory capabilities (Mehta et al., 2020). Similarly, flexibility and bending play a direct role in the flow-sensing abilities of cilia (Nauli et al., 2008; AbouAlaiwi et al., 2009; Goetz et al., 2014). It is possible that these mechanosensors are therefore able to distinguish between LF and DF via their conformational flexibility and trigger appropriate and differing responses. Additionally, and as discussed above, GPCRs and Piezo1 are able to sense both LF and DF, and can trigger integrin activity under DF or eNOS activity under LF conditions (Albarrán-Juárez et al., 2018). How both shear stress profiles are sensed in this case is not yet known, but changes in Piezo1 structure or the recently shown mechanosensitive helix 8 motif of GPCRs (Erdogmus et al., 2019) could play a role in the abilities of these mechanosensors to sense both LF and DF. Understanding these fundamental differences in mechanoreceptors will shed further light on the spatial regulation of endothelial responses to differential flow.

The KLF transcription factors are master regulators of endothelial shear stress response and play a pivotal role in regulating the anti-inflammatory signalling in areas of LF (SenBanerjee et al., 2004; Dekker et al., 2002) (Fig. 2). KLF2 is activated by LF and displays reduced activity under DF (Chien, 2008; Dekker et al., 2002, 2005; Parmar et al., 2006), and the homologous KLF4 plays an analogous role regulating LF-dependent anti-inflammatory signalling (McCormick et al., 2001; Hamik et al., 2007). Two upstream modulators of KLF2, MEK5 (MAP2K5) and ERK5 (MAPK7), were recently found to be regulated by shear-induced mitochondrial reactive oxygen species (ROS) production, leading to transcriptional induction of *KLF2* (Coon et al., 2022) (Fig. 2). In the aorta, global knockout of PINK1, a key regulator of mitophagy, reduced ERK5 nuclear localisation and *KLF2* expression in the descending aorta, whereas no difference was noted in the inner arch (Coon et al., 2022). Collectively, this study indicates how spatial responses to differing flow profiles of the descending aorta and aortic arch may arise through mitophagy induced by LF and DF (Fig. 2), and how this induces or supresses KLF2 expression, respectively, via the MEK and ERK pathway.

Downstream of mechano-induced site-specific signalling in the aorta, inflammatory and cell adhesion molecules, namely, VCAM1 and ICAM1, are expressed in DF-exposed regions (Iiyama et al., 1999) owing to suppressed anti-inflammatory activity of the KLFs (SenBanerjee et al., 2004; Parmar et al., 2006), increased YAP/TAZ nuclear localisation (Wang et al., 2016a,b) and increased NFκB activity (Khachigian et al., 1995; Hajra et al., 2000) (Fig. 2). The spatial localisation of VCAM1 was recently mapped across the aorta by whole-mount and whole-tissue immunofluorescence coupled with single-cell RNA sequencing (scRNAseq), and indicated strong expression in a specific subcluster of ECs in DF-exposed regions of the aorta (Kalluri et al., 2019). Interestingly, scRNAseq also demonstrated that the anti-angiogenic cell surface receptor CD36 was not expressed in VCAM1-expressing ECs (Fig. 2), which was validated by *in situ* staining. This study highlighted the existence of heterogeneous ECs across the aorta that exhibit specific signalling tied to their spatial location (Kalluri et al., 2019).

### YAP/TAZ

Within the inner aortic arch, the mechanosensitive transcriptional co-activators YAP (or YAP1) and TAZ (WWTR1) are active due to the DF in this region (Fig. 2). YAP/TAZ are regulated in part by phosphorylation status and are inactive when phosphorylated, leading to proteasomal degradation. Conversely, active YAP/TAZ are dephosphorylated and translocate to the nucleus (Dupont et al., 2011). In ECs, DF triggers YAP/TAZ activity, nuclear localisation and transcription of target genes, whereas long-term LF leads to inhibition of YAP/TAZ activity. However, exposing static ECs to flow leads to transient YAP activity, which diminishes over time if flow rates are kept constant. This flow-induced YAP activity was shown to be regulated by cytoskeletal dynamics and binding of YAP to angiomotin within the cell-cell junctions (Nakajima et al., 2017). GPCR signalling and integrin β1 (ITGB1) regulate YAP/TAZ activity in the aorta, increasing YAP/TAZ activity in the inner arch but decreasing it in the descending aorta (Wang et al., 2016b). Although the roles of other forces, such as stiffness, in activating YAP/TAZ have been investigated in depth, how DF and LF regulate YAP localisation and activity remains poorly understood. Stiffness regulates nuclear pore complex conformation, allowing the shuttling of YAP to the nucleus under increasing ECM stiffness (Andreu et al., 2022). Whether shear stress leads to similar nuclear pore complex conformational changes to regulate YAP/TAZ localisation remains to be shown.

Overall, several surface and junctional proteins have been shown to act as direct mechanical sensors of shear stress, which indicates how spatially distinct signalling is tuned in the endothelium of the aorta. Although recent work has shed significant light on these mechanisms, our understanding remains far from complete.

## Chromatin and epigenetics

**Fig. 3.**
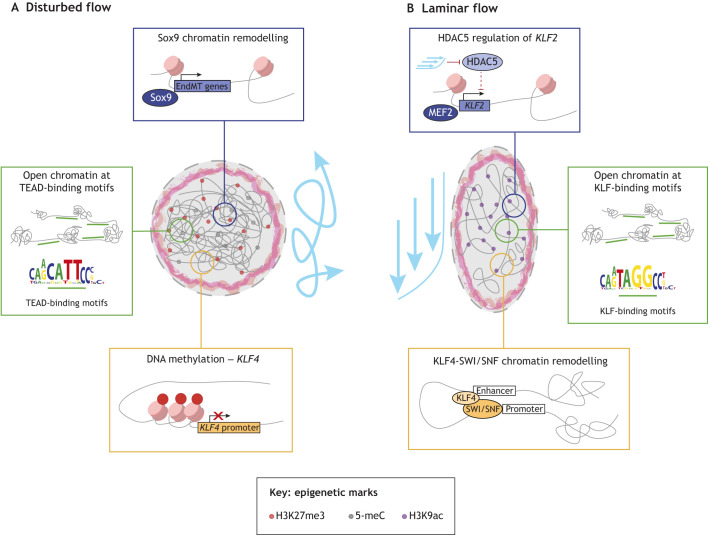
**The effects of the shear stress profile on the epigenetic and chromatin states of endothelial cells.** We propose that current evidence indicates that disturbed flow leads to repressed, methylated and condensed chromatin, in contrast to the open, decondensed chromatin under laminar flow conditions. We also note that many of these mechanisms have not yet been temporally resolved, although we know that mechanosensitive epigenetic and chromatin states are modulated over time, depending on whether the initial force is maintained (Miroshnikova et al., 2017). (A) Under disturbed flow, endothelial cells exhibit rounded nuclear morphology and display increased repressive histone modifications, such as the histone H3 lysine 27 trimethylation (H3K27me3) mark catalysed by the methyltransferase EZH2. Simultaneously, disturbed flow increases the accessibility of TEAD-binding motifs (green box) and the binding of Sox9 to promotors of endothelial-mesenchymal transition (EndMT)-related genes (blue box). Disturbed flow also increases DNMT1-dependent DNA methylation (orange box). (B) Under laminar flow conditions, chromatin is more open and accessible, mainly due to increased levels of histone H3 lysine 9 acetylation (H3K9ac). This is because laminar flow inhibits histone deacetylase (HDAC) activity, which leads to MEF2-driven expression of the anti-inflammatory atheroprotective KLF2 (blue box). Beyond KLF2 expression, laminar flow promotes open, decondensed chromatin around KLF-binding motifs in the promotors of a number of genes (green box), as well as increased enhancer-promotor interactions facilitated by the KLF4-SWI/SNF complex (orange box). 5-meC, 5-methycytosine.

The transcriptional response to shear stress is well documented, rendering the impact of mechanical force in modulating chromatin dynamics and epigenetic changes an area of great interest. Initial studies showed that shear stress regulates histone acetylation and phosphorylation, and alters histone acetyltransferase activity and expression (Illi et al., 2003). Following these studies, KLF2 was shown to be modulated by flow-dependent epigenetic changes within the *KLF2* promotor (Wang et al., 2010). In this study, it was shown that LF induced the dissociation of histone deacetylase (HDAC) 5 from the KLF2 promotor and allowed MEF2 (or MEF2C) transcription factor binding to drive *KLF2* expression (Fig. 3B, blue box). Furthermore, Lee et al. (2012) showed that DF leads to nuclear accumulation of HDACs 1, 2, 3, 5 and 7 in ECs of the inner arch and regulates flow-dependent expression of KLF2 and VCAM1 and EC proliferation.

Histone modifications are associated with specific flow profile patterns. The histone methyltransferase EZH2, which trimethylates histone H3 at lysine 27 (H3K27), was shown to be flow sensitive and exhibited increased activity in the aortic arch compared to in the descending aorta, suggesting flow pattern-specific epigenetic regulation of histone H3 methylation (Xu et al., 2018b). In agreement with this, global H3K27 trimethylation (H3K27me3) levels were increased in the inner arch compared to those in the descending aorta. The authors found that histone markers of decondensed chromatin, such as acetylation of histone H3 at lysine 9 (H3K9ac), increased under LF and were enriched in the descending aorta versus at the inner arch. Additionally, decondensation of chromatin by treatment with a HDAC inhibitor resulted in faster alignment of ECs to LF (Danielsson et al., 2022b). Moreover, in the skin epidermis, histone modifications are force responsive and subsequently protect against the DNA-damaging effects of mechanical stretching by modulating chromatin condensation (Nava et al., 2020). In line with these findings, Danielsson et al. (2022b) showed that inhibition of chromatin decondensation in ECs induced DNA damage under LF.

DNA methylation at CpG islands within gene promotors represses transcription. Earlier studies showed that DF led to upregulation of the methyltransferase DNMT1, leading to global DNA hypermethylation, a proinflammatory EC profile and atherosclerosis (Dunn et al., 2014; Zhou et al., 2014). DNMT1 also regulates *KLF4* promotor methylation under DF to permit the expression of the pro-inflammatory cytokine *CCL2*, which would otherwise be supressed under LF (Jiang et al., 2014) (Fig. 3A, orange box). Davies et al. (2014) suggested that NFκB, which, as we previously discussed, is active in ECs exposed to DF, can recruit DNMTs to specific genetic loci. Overall, these studies suggest that histone modifications and DNA methylation in ECs are flow specific, with repressive methylation of histones and DNA enriched under DF and more open active chromatin under LF conditions. As pointed out by Danielsson et al. (2022b), whether these flow-induced epigenetic modifications alter nuclear stiffness remains an interesting open question for the field.

Developments in single-cell sequencing have given spatial resolution to the influence of shear stress on epigenetic regulation of the endothelium *in vivo*. A multi-omics approach by He et al. (2019) showed that, in ECs exposed to pulsatile laminar and oscillating flow, KLF4 is required for LF-induced H3K27 acetylation, chromatin accessibility and, consequently, transcription of the cytosolic calcium channel gene *ITPR3* to maintain vascular homeostasis. Tsaryk et al. (2022) similarly showed that exposing ECs to LF for 6 h *in vitro* remodelled enhancers, particularly those at KLF-binding sites, which was validated *in vivo*. This study also identified LF-induced repression of enhancers by reducing transposase accessibility, H3K27ac and enrichment of ETV2/ETS-binding sites. Further validating the link between shear stress and endothelial chromatin dynamics, Andueza et al. (2020) showed that DF indeed alters chromatin accessibility, broadly both opening and closing distinct regions, *in vivo*. The authors ligated murine carotid arteries to induce DF in vessels that would otherwise be exposed to LF, and integrated scRNAseq analyses and single-cell sequencing assay for transposase-accessible chromatin (scATACseq) analyses of the ECs. These analyses indicated broad EC heterogeneity under LF and that exposure to DF initiated pro-inflammatory reprogramming through the modulation of KLF- and TEAD-binding motif accessibility (Fig. 3A,B, green boxes). This study, as Tsaryk et al. (2022) discussed above, also suggested that enhancers as well as cis-regulatory elements are altered by differing flow patterns. Additionally, Andueza and colleagues also showed that enhancer-like elements around the KLF4 promotor may exhibit increased interactions under LF compared to under DF, suggesting that flow-induced chromatin accessibility may regulate *KLF4* transcription (Andueza et al., 2020). This work was furthered by a recent study by Moonen et al. (2022), who showed that as well as coordinating an atheroprotective transcriptional programme under LF, KLF4 also modulates enhancer accessibility by interacting with the SWI/SNF chromatin-remodelling complex to regulate the expression of flow-responsive genes (Fig. 3B, orange box). These authors also showed that KLF4 regulated non-coding regions, including one in an enhancer of the mechanosensor JCAD, which previous genome-wide association studies identified as important in the development of atherosclerosis and coronary artery disease (Douglas et al., 2020; Xu et al., 2019).

The developmental transcription factor Sox9 was also implicated in chromatin remodelling of ECs. Fuglerud et al. (2022) analysed the scATACseq dataset from the Andueza et al. (2020) paper discussed above to show that that DF-exposed ECs express increased levels of *Sox9*, and that Sox9 remodels EC chromatin during EndMT (Fig. 3A, blue box). Interestingly, recent work has also shown that YAP regulates chromatin accessibility by regulating SOX9 expression in induced pluripotent stem cell (iPSC)-derived ECs (Liu et al., 2023). Indeed, YAP/TAZ were also shown to mechano-modulate chromatin dynamics outside of the endothelial context, through co-option of SWI/SNF in cancer under conditions of altered ECM stiffness (Chang et al., 2018). In addition to this, Bondareva et al. (2019) showed that exposing ECs to oscillatory shear stress changed YAP/TAZ-responsive regulatory elements upstream of certain target genes, indicating a role for YAP/TAZ binding for these oscillatory flow-responsive elements. This suggests that chromatin dynamics, histone modifications and enhancers are modulated by different types of shear stress in a YAP/TAZ-dependent manner. However, intriguing questions remain, in particular, the *in vivo* evaluation of the role of YAP/TAZ role and their ability to shape EC chromatin in different regions of the aorta. Collectively, these studies indicate the intricate nature of gene regulation through coordination of multiple components and start to unpick the role of shear stress in shaping these genomic associations for appropriate flow-mediated gene expression.

## Endothelial nuclear mechanosensing

As the largest and stiffest organelle, the nucleus plays an important role in mechanosensing and mechanotransduction. The relevance for the endothelium as a paradigm for studying nuclear mechanosensing was indicated by experiments in which isolated nuclei were exposed to shear stress. This changed the nuclear lamina (lamin A/C, encoded by *LMNA*), leading to stiffer nuclei (Swift et al., 2013; Deguchi et al., 2005).

Because of the thin, flat morphology of ECs, the nucleus is elevated above the rest of the cell body and thus is primarily exposed to shear stress, creating hydrodynamic drag (Tkachenko et al., 2013). Tkachenko et al. (2013) suggested that this drag on the nucleus changes the cytoskeleton to confer tension on the mechanosensory junctional complex and trigger flow-responsive signalling, dictating polarity and positioning both the nucleus and the microtubule-organizing centre. Shear stress can increase stiffness, decrease height and align nuclei parallel to the direction of flow (Dahl et al., 2008). In this capacity, nuclei align or remodel themselves to limit the exposure to mechanical force in order to protect genomic integrity. These alignment and remodelling responses were shown in the seminal paper by Nava et al. (2020) in epidermal cells undergoing stretch and were subsequently confirmed in flow-exposed ECs (Danielsson et al., 2022b).

The nucleus consists of an inner and outer nuclear membrane (INM and ONM, respectively) consisting of embedded protein complexes that bridge the cytoskeleton to the ONM and INM and directly tether chromatin. The linker of the nucleoskeleton and cytoskeleton (LINC) complex serves as a molecular bridge between the cytoskeleton, nuclear membrane and the nuclear lamina (Haque et al., 2006; Crisp et al., 2005). The nuclear lamina, composed of A- and B-type lamin, forms a network within the INM. As such, it is in direct contact with chromatin and has been shown to adapt to mechanical force (Guilluy et al., 2014). A host of other nuclear envelope transmembrane proteins, including emerin, serve as transducers of mechanical force (Foisner and Gerace, 1993; Guilluy et al., 2014; Cai et al., 2001; Manilal et al., 1996; Nagano et al., 1996).

Individual components of the nuclear membrane determine the appropriate response of ECs to mechanical force. The LINC components nesprin 1 (SYNE1) and 2 (SYNE2) regulate EC and nuclear shape as well as angiogenic capacity (King et al., 2014). Under cyclic strain, nesprin 1 was required for EC alignment perpendicular to the direction of stretching (Chancellor et al., 2010; Anno et al., 2012). Additionally, nesprins regulate nuclear height and shape. The association of the cytoskeleton to nesprin, as well as tension across the nucleus, lower nuclear height and perturb the actomyosin organisation of ECs (Chancellor et al., 2010). Nesprin 3 (SYNE3) affects the positioning of the microtubule-organizing centre, which regulates the cytoskeleton, nuclear shape and the polarisation of ECs under shear stress (Morgan et al., 2011). Moreover, expression of the dominant-negative form of the KASH domain of nesprin 1 (DN-KASH), which inhibits the binding of the cytoskeleton to the nuclear membrane via displacement of all nesprins, impairs EC mechanosensing (Denis et al., 2021). In this study, the reduced FAK activity, adhesion, cell attachment and wound healing suggest bidirectional signalling from the nuclear membrane to the mechanosensors on the surface of the cell. Interestingly, under LF, the cell bodies and nuclei of DN-KASH-expressing ECs were still able to align parallel to flow (Denis et al., 2021).

## A continuous connection to the nucleus

**Fig. 4.**
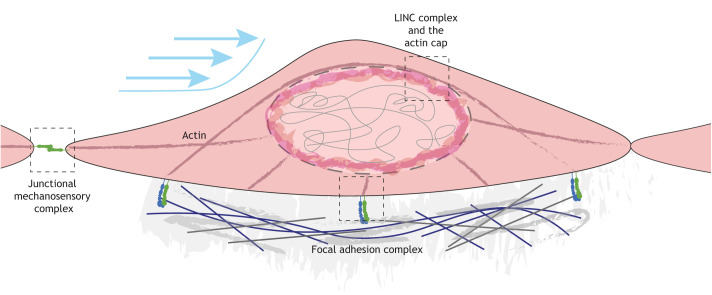
**Contiguous connections bridge extracellular mechanosensors and the nuclear membrane via the actin cytoskeleton.** Shear flow is sensed at the cell-cell junctions and the cell membrane, and by integrin-based focal adhesions. Continuous connections between these mechanosensory and transducing elements and the nuclear membrane via the cytoskeleton and the linker of the nucleoskeleton and cytoskeleton (LINC) complex have been described. Emerging evidence suggests that these connections may inform flow-induced chromatin dynamics that determine how cells respond to shear stress.

The hypothesis of a direct contiguous connection that links cell surface mechanosensors to the nucleus to inform genomic events was originally described in ECs by Maniotis et al. (1997). This has since been confirmed with the identification of an actin cap that links focal adhesions to the LINC complex (Chambliss et al., 2013). The actin cap develops in response to the fluid shear stress and mediates force transmission from zyxin under low shear stress and from talins under higher shear stress (Chambliss et al., 2013). Recent work from the Bautch laboratory indicated communication from the LINC complex to junctions via a microtubule-SUN1 connection to modulate endothelial barrier function (Buglak et al., 2023). Work from our own laboratory shows that the junctional molecule AmotL2 binds VE-cadherin and p120 catenin in a complex to link SUN2 via the actin cytoskeleton, a connection that is required for shear stress-mediated EC alignment (Zhang et al., 2023). A follow-up preprint from our laboratory indicates that AmotL2 is required for lamin A expression and that AmotL2 loss reduces chromatin accessibility of the *YAP* promotor via increased repressive H3K27me3 marks to silence *YAP* transcription in ECs. This work also indicates that YAP/TAZ are required for the nuclear actin cap in ECs of the thoracic aorta (Mannion et al., 2023 preprint), in agreement with Coleman et al. (2020) showing that YAP/TAZ were required for EC alignment via their transcriptional activation of the GTPase activator gene *ARHGAP18* and via modulation of actin dynamics (Coleman et al., 2020). Interestingly, YAP also plays a central role in regulating the actin cap in epithelial cells by directly binding to the promotors of *LMNB1* and *ACTR2* (Sladitschek-Martens et al., 2022). Whether YAP similarly regulates components of the nuclear actin-binding proteins in ECs to control their alignment remains to be shown. Overall, these emerging studies suggest a continuous link between cell-cell junctions and the nuclear membrane that informs nuclear dynamics and endothelial (and epithelial) responses to mechanical force (Fig. 4).

Cooperation between mechanosensors has been documented by numerous laboratories, showing that junctional mechanosensing can lead to the activation of integrins and focal adhesions. We have previously discussed work on other mechanosensors such as Piezo1 and the cooperation between junctions (Chuntharpursat-Bon et al., 2023) and GPCRs (Albarrán-Juárez et al., 2018). Precisely how these mechanosensors cooperate with the nucleus to inform wider transcriptional adaptation to force is less well understood. The emerging evidence discussed above indicates that junctions cooperate with the nucleus in sensing mechanical forces via the cytoskeletal network (Chambliss et al., 2013; Buglak et al., 2023; Zhang et al., 2023). Changes in nuclear shape (Jung et al., 2012) and downstream changes to chromatin are observed in endothelial-specific deletions of the GPCR gene *S1pr1* (Engelbrecht et al., 2020); however, the link between GPCRs and the nucleus is not clear. Similarly, whether cilia and the glycocalyx are implicated in regulating the nucleus remains unclear. Further research is therefore needed to understand the importance of primary mechanosensors in bidirectional communication with the nucleus via other signalling networks.

## Consequences of impaired endothelial mechanosensing

### Laminopathies: progeria

Mutations in components of the nuclear lamina cause laminopathies, a diverse group of rare diseases that include white matter disorders, skeletal and cardiac muscle dystrophies and accelerated ageing (progeria). Laminopathies are linked to impaired mechanosensing, which has implications for the cardiovascular system (Goldman et al., 2004; Olive et al., 2010; Baker et al., 1981).

Hutchinson–Gilford progeria syndrome (HGPS) is caused by a single point mutation in *LMNA*, which leads to a truncated variant of lamin A known as progerin (De Sandre-Giovannoli et al., 2003). This mutation activates a cryptic splice site that ultimately results in progerin being permanently farnesylated and subsequently unable to be processed into mature lamin A (Fong et al., 2004). The nuclei of patients with HGPS therefore exhibit abnormal morphology, increased stiffness and altered chromatin and gene expression (Dahl et al., 2006, 2008). Osmanagic-Myers et al. (2019) developed a mouse model of HGPS via endothelial-specific expression of progerin. This led to cardiovascular disorders, phenocopying the human disease profile. As well as cardiac thickening and fibrosis, the ECs in the descending aorta of these HGPS mice were unable to align in the direction of flow. Exposing the progerin-expressing ECs to short-term shear flow *in vitro* showed that their nuclei failed to align, had upregulated expression of the INM components SUN1/2 and exhibited altered emerin activity (Osmanagic-Myers et al., 2019). Interestingly, longer-term (24-72 h) exposure of progerin-expressing ECs to shear stress also impaired alignment, altered nuclear morphology and induced cell detachment. These effects were phenocopied by depletion of the protease ZMPSTE24, which targets the farnesylation of wild-type lamin A and therefore mimics progerin expression, and were rescued by treatment with the farnesylation inhibitor lonafarnib (Danielsson et al., 2022a). Another study of endothelial-specific expression of progerin showed that destabilisation of the deacetylase Sirt7 led to accelerated ageing, atherosclerosis and inflammation (Sun et al., 2023). Further work using iPSC-derived ECs from HGPS patients showed impaired nuclear orientation and roundness under shear flow. Additionally, compared to wild-type ECs, exposing these HGPS iPSC-derived ECs to shear stress reduced the upregulation of *KLF2* and *NOS3*, indicating that flow-induced transcriptional responses are impaired in progerin-expressing ECs (Atchison et al., 2020). Defects in *Nos3* expression were also evident in the *in vivo* EC-specific HGPS model described above, which were due to decreased MRTF signalling (Osmanagic-Myers et al., 2019). Given the causative association between nuclear architecture and genomic output, interesting questions remain as to the effects of shear stress on EC chromatin structure in progeria and how these affect shear stress-induced reprogramming of the endothelium to drive the disease phenotype.

### Atherosclerosis

**Fig. 5.**
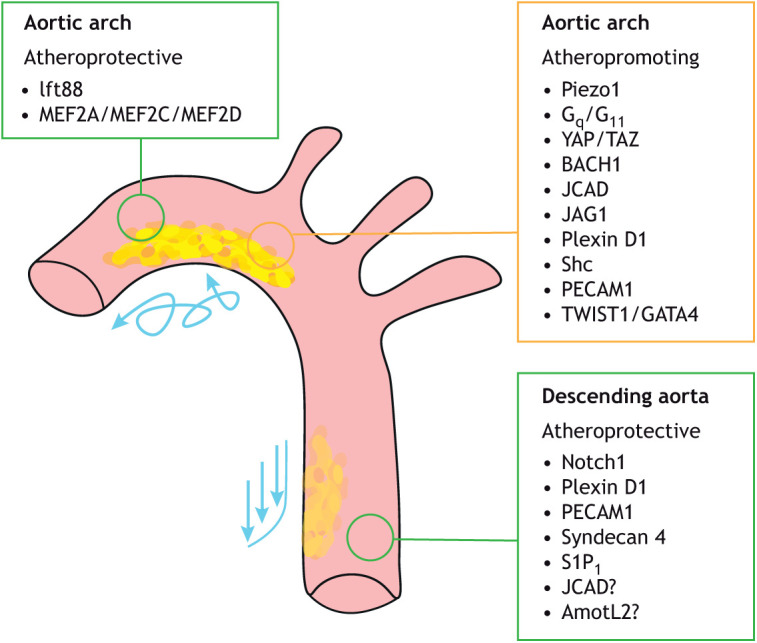
**Endothelial mechanosensors and mechanotransducers and their role in atherosclerosis.** Studies on the role of the mechanosensors and mechanotransducers listed here have used inducible endothelial-specific knockout mouse models (Dinsmore and Reiter, 2016; Albarrán-Juárez et al., 2018; Li et al., 2019; Jia et al., 2022; Xu et al., 2019; Douglas et al., 2020; Souilhol et al., 2023; Mehta et al., 2020, 2023; Goel et al., 2008; Mahmoud et al., 2019; Mack et al., 2017; Baeyens et al., 2014; Galvani et al., 2015; Lu et al., 2021) to discover how these either promote or protect against atherosclerosis. To further investigate the compounding effects of genetic predisposition and metabolism, some of these endothelial-specific knockout mice were crossed with strains that exhibit accelerated atherosclerotic development, such as *Apoe*-knockout (Zhang et al., 1992) and *Ldlr*-knockout (Ishibashi et al., 1993) mice. These studies have highlighted how the mechanosensors of disturbed or laminar flow act as either atherogenic (orange box) or atheroprotective (green boxes) factors.

Regions of the aorta exposed to DF, such as the inner aortic arch, are predisposed to inflammation due to decreased anti-inflammatory and increased pro-inflammatory signalling and adhesion molecules, as discussed above. Added risk factors, such as poor diet and genetic predisposition, can trigger the influx of immune cells, accumulation of lipids and plaque development in these already insulted endothelial niches, leading to atherosclerotic lesions that pose significant risk to further pathological phenomena. A precursor step in the development of atherosclerosis is the reprogramming of the endothelium to a more mesenchymal phenotype in EndMT via the upregulation of transcription factors such as TWIST1 (Mahmoud et al., 2019), which is initiated by DF (Fig. 5). For further reading, EndMT is reviewed extensively in Bischoff (2019), Souilhol et al. (2018) and Chen et al. (2020). This DF-driven reprogramming is facilitated by chromatin dynamics (Andueza et al., 2020; Tamargo et al., 2023; Fuglerud et al., 2022; Jiang et al., 2018) and mechanosensory pathways (Mehta et al., 2023; Mahmoud et al., 2019), which we have touched upon already.

Generally, impaired mechanosensing and alignment to LF stimulates EC inflammation. This is supported by evidence showing that deletion of the mechanosensors PECAM1, plexin D1, syndecan 4 (SDC4) and Notch1 leads to atherosclerotic plaque development in normally atheroprotected regions of the descending aorta (Fig. 5). Plexin D1 regulates the atheroprotective transcription factors KLF2 and KLF4 and protects against inflammation and atherosclerosis in the descending aorta where blood flow is laminar (Mehta et al., 2020). Simultaneously, endothelial expression of plexin D1 in the aortic arch promotes plaque development, as endothelial deletion of plexin D1 reduces the plaque burden of the aortic arch (Mehta et al., 2020). Another member of the junctional mechanosensory complex, PECAM1, exhibits a similar site-specific atheroprotective role in the descending aorta and an atherogenic role in the arch (Goel et al., 2008). Endothelial expression of the transmembrane protein syndecan 4 was also found to be atheroprotective, as its deletion caused impaired EC alignment and plaque development specifically in atheroprotected regions (Baeyens et al., 2014). Furthermore, ECs of the descending murine aorta exhibit high Notch1 activity, which was required for their alignment to shear stress in a KLF2-independent manner. Here, flow induces the cleavage and activation of Notch1 to initiate an anti-inflammatory transcriptional program. Deletion of *Notch1* was in turn shown to promote atherosclerosis in regions usually ath eroprotected by LF (Mack et al., 2017).

Interestingly, cilia play a protective role in the aortic arch. Deleting the *Ift88* gene, which is required for ciliogenesis, specifically in ECs of the arch enhances plaque development (Dinsmore and Reiter, 2016) (Fig. 5). As mentioned above, some mechanosensory elements, such as plexin D1 and PECAM1, fall into both categories – having protective roles in the descending aorta and atherogenic roles in the aortic arch – indicating that these mechanosensors dictate site-specific endothelial responses to flow. However, a number of mechanosensory and mechanotransductory mediators are atherogenic in regions such as the inner aortic arch (Fig. 5).

Among these mechanosensors, YAP/TAZ play an important role in facilitating the proinflammatory and atherogenic phenotype of the EC of the aortic arch (Wang et al., 2016a,b) (Fig. 5). LF promotes integrin-Gα_13_ interactions and supresses YAP via reduced RhoA activity (Wang et al., 2016b), whereas DF promotes active nuclear YAP via the integrin α5β1 pathway and c-Abl kinase (or ABL1) to drive downstream *ICAM1* and *VCAM1* expression and atherosclerosis (Li et al., 2019). The YAP/TAZ target CCN1 promotes atherosclerosis by shear stress-induced binding to integrin α6β1 to trigger downstream NFκB activity (Hsu et al., 2019). Interestingly, a recent study identified the transcription factor BACH1 as a mechanosensor that translocates to the nucleus under DF, where it acts as a binding partner of YAP (Jia et al., 2022). Under DF, the BACH1-YAP pro-inflammatory transcriptional programme drives the development of atherosclerosis. Additionally, BACH1 was found to bind to the *YAP* promotor and directly activate *YAP* transcription.

The mechanosensor JCAD was identified in genome-wide association studies of patients with coronary artery disease (Erdmann et al., 2011), and *Jcad* deficiency led to reduced development of atherosclerosis in *Apoe*-knockout mice (Douglas et al., 2020; Xu et al., 2019) (Fig. 5). Xu et al. (2019) also showed that, in human ECs, JCAD depletion repressed YAP activity by stabilising its interaction with the cytoskeletal modulator TRIOBP to block its entry into the nucleus. This prevented the expression of its downstream atherogenic target *CCN1*, confirming that JCAD promotes endothelial dysfunction, inflammation and atherosclerosis (Xu et al., 2019). Although reduced VCAM1 and ICAM1 levels were noted in the aortic arch, in line with an atheroprotective phenotype, Douglas et al. (2020) observed increased VCAM1 expression in the descending aorta of *Jcad*-knockout mice, suggesting that JCAD may also have site-specific roles in promoting atherosclerosis.

Both cell-cell junctions and cell-cell communication play crucial roles in maintaining the endothelium. The Notch pathway is a conserved cell-cell communication pathway with broad implications. Despite the atheroprotective role of Notch1 described by Mack et al. (2017), Souilhol et al. (2023) showed that DF activates JAG1-Notch4 signalling in the inner aortic arch. This axis supresses proliferation and vascular repair, and endothelial-specific deletion of JAG1 protected against atherosclerosis of the arch (Fig. 5), highlighting the divergent roles of Notch signalling in EC mechanosensing. The signal transducer Shc is required for flow-dependent Notch signalling (Sweet et al., 2013). This is triggered by the mechanosensory activity of Alk5, which activates downstream SMAD signalling and inflammation, leading to atherosclerotic plaque development in the aortic arch (Mehta et al., 2023).

We already discussed how Piezo1 promotes atherosclerotic development in the inner aortic arch through atherogenic NFκB signalling (Albarrán-Juárez et al., 2018) (Fig. 5). Piezo1 is also seemingly required for the atheroprotective eNOS activity induced by LF. However, plaque development was not noted in the descending aorta of *Piezo1*-knockout mice, which could be due to FAK-mediated NFκB activation by DF (Albarrán-Juárez et al., 2018).

Overall, these studies show that a number of mechanosensing apparatuses, from primary sensors to downstream signalling components of mechanotransduction, are implicated in the development of atherosclerosis. The differential role of these components in sensing DF and LF indicates that regulation of EC phenotype requires an intricate orchestration of these components to suppress pathological development, either through measured response to LF or suppressed pro-inflammatory signalling initiated by DF.

### Aneurysms

Aneurysms are caused by vascular instability, where the ability of the vascular wall to contain outward forces results in bulging and ballooning of the vessel. Although the endothelium primarily senses shear flow, the role of the underlying intima, composed of the supporting parenchymal cells and vascular smooth muscle cells, in aneurysm development is far better understood. Indeed, impaired mechanosensing and genetic factors are more often associated with dysfunction of vascular smooth muscle cell contractility (Chou et al., 2023). However, the changes in vessel architecture due to the bulging of aneurysms induce changes to flow dynamics, which affect the endothelium to potentiate disease development and aneurysm growth (Baeriswyl et al., 2019; Levitt et al., 2019; Harloff et al., 2010). For example, bulge-induced DF reduces the capacity of ECs to regulate oxidative stress, which leads to ROS, increased inflammation, immune influx and the subsequent degradation of the ECM, which is a key contributor to the onset of aneurysms (Lowis et al., 2023). EC-specific deletion of GTP-cyclohydrolase I (GCH1), an enzyme that prevents eNOS uncoupling, renders mice more susceptible to abdominal aortic aneurysm (AAA) upon angiotensin II challenge (Chuaiphichai et al., 2018). Additionally, onset of murine aneurysms upon chronic subcutaneous infusion of angiotensin II, which is a well-established model, requires endothelial expression of type 1A angiotensin II receptor (At1a or Agtr1), whereas expression of the same receptor in vascular smooth muscle cells is redundant (Rateri et al., 2011). Despite the lack of studies outlining EC-specific mechanosensing defects in aneurysm development, emerging evidence is beginning to highlight the importance of this mechanosensing route.

Further emphasising the importance of the ECM, Marfan syndrome, defined by mutations in the ECM component fibrillin-1 (FBN1), presents with an increased risk of aortic aneurysm (Pereira et al., 1997; Dietz and Pyeritz, 1995). Introducing the Marfan-specific mutation *Fbn1^C1041G^* specifically into murine ECs impaired their alignment within both the ascending and descending aorta, and perturbed junctional stability under LF (Mieremet et al., 2022). The same study showed that treatment with resveratrol restored EC alignment, phospho-eNOS expression and vascular structure, effectively rescuing the phenotype. Beyond the ECM, impaired junctional mechanosensing by EC-specific AmotL2 deletion also leads to misaligned aortic ECs, reduced RhoA activity and upregulation of inflammatory molecules (Zhang et al., 2023). This manifests in the development of AAA in male mice and highlights the importance of the above-described junctional-nuclear connection via the cytoskeleton for maintaining vascular homeostasis.

The global deletion of Notch1 protects against AAA (Hans et al., 2012). Recent work has shown that blocking Notch signalling with the γ-secretase inhibitor *N*-[*N*-(3,5-difluorophenacetyl)-L-alanyl]-*S*-phenylglycine *t*-butyl ester (DAPT) reduces AAA incidence in the angiotensin II-induced mouse model (Cheng et al., 2023). Corroborating these studies, scRNAseq of the murine aortic endothelium found that, of the transcripts with known involvement in familial thoracic aortic aneurysm and dissection, *Notch1* was the only one exclusively expressed in ECs (Kalluri et al., 2019). Given the known role of endothelial Notch1 in mechanosensing of the aorta (Mack et al., 2017), it is tempting to speculate that EC-specific Notch1 signalling may predispose the descending aorta to aneurysms.

## Future perspectives

Collectively, the studies we have discussed here show that ECs sense DF and LF using a variety of mechanosensors, and that these forces are transduced and integrated by modulation to chromatin accessibility and transcriptional output.

Although changes to chromatin and transcription under differing conditions of flow have now been demonstrated *in vitro* and *in vivo*, the temporal dynamics of these modifications remain to be resolved. Disparity in time scales between studies, where exposure to transient flow can range from seconds to days *in vitro* and from days to weeks *in vivo*, may play a role in the outcome and interpretation of results. Furthermore, how the onset of flow within the developing mammalian aorta informs morphological adaption and chromatin organisation is still poorly understood, particularly with respect to the region-specific flow patterning displayed in the aorta.

Important questions also remain regarding chromatin dynamics in response to specific flow profiles. For instance, how does the primary flow-sensing apparatus at the junctions and cell membrane influence nuanced epigenetic regulation in the nucleus? Emerging evidence on the effects of flow on the nucleus in laminopathies have highlighted a role for the nuclear envelope in EC mechanosensing, and studies have begun to uncover the role of specific components. However, important questions remain as to whether chromatin and epigenetic mechanisms are affected by impaired nuclear mechanosensing. Recent work in cutaneous squamous cell carcinoma showed a pathway from the adhesome to the nuclear membrane that, when disrupted, led to chromatin reorganisation and its repositioning to the nuclear periphery and concurrent silencing of gene expression (Chee et al., 2023). Similar studies delineating signal transduction from mechanosensors to the nuclear membrane and the effects this has on chromatin localisation and the nuclear periphery in ECs exposed to differential flow will provide important insight into mechanically regulated EC biology.

Finally, the use of highly detailed existing and future datasets from scRNAseq, chromatin immunoprecipitation followed by sequencing (chIP-seq), ATACseq and Hi-C are likely to reveal further details on how signalling from sensors and transducers to the nuclear membrane informs intranuclear dynamics. Applying these advanced omics technologies will facilitate the delineation of spatially and, in the case of the aorta, mechanically unique mechanisms that program the endothelium. These will hopefully lead to important insights that could shape our understanding of vascular disease settings such as atherosclerosis, aneurysms and laminopathies.

## References

[DMM050361C1] Aboualaiwi, W. A., Takahashi, M., Mell, B. R., Jones, T. J., Ratnam, S., Kolb, R. J. and Nauli, S. M. (2009). Ciliary polycystin-2 is a mechanosensitive calcium channel involved in nitric oxide signaling cascades. *Circ. Res.* 104, 860-869. 10.1161/CIRCRESAHA.108.192765 19265036 PMC3085025

[DMM050361C2] Aitken, C., Mehta, V., Schwartz, M. A. and Tzima, E. (2023). Mechanisms of endothelial flow sensing. *Nat. Cardiovasc. Res.* 2, 517-529. 10.1038/s44161-023-00276-0 39195881

[DMM050361C3] Albarrán-Juárez, J., Iring, A., Wang, S., Joseph, S., Grimm, M., Strilic, B., Wettschureck, N., Althoff, T. F. and Offermanns, S. (2018). Piezo1 and Gq/G11 promote endothelial inflammation depending on flow pattern and integrin activation. *J. Exp. Med.* 215, 2655-2672. 10.1084/jem.20180483 30194266 PMC6170174

[DMM050361C4] Altschul, R. (1954). *Endothelium: Its Development, Morphology, Function, and Pathology.* New York: The MacMillan Company.

[DMM050361C5] Andreu, I., Granero-Moya, I., Chahare, N. R., Clein, K., Molina-Jordán, M., Beedle, A. E. M., Elosegui-Artola, A., Abenza, J. F., Rossetti, L., Trepat, X. et al. (2022). Mechanical Force Application to the Nucleus Regulates Nucleocytoplasmic Transport. *Nat. Cell Biol.* 24, 896-905. 10.1038/s41556-022-00927-7 35681009 PMC7614780

[DMM050361C6] Andueza, A., Kumar, S., Kim, J., Kang, D.-W., Mumme, H. L., Perez, J. I., Villa-Roel, N. and Jo, H. (2020). Endothelial reprogramming by disturbed flow revealed by single-cell RNA and chromatin accessibility study. *Cell Rep.* 33, 108491. 10.1016/j.celrep.2020.108491 33326796 PMC7801938

[DMM050361C7] Anno, T., Sakamoto, N. and Sato, M. (2012). Role of nesprin-1 in nuclear deformation in endothelial cells under static and uniaxial stretching conditions. *Biochem. Biophys. Res. Commun.* 424, 94-99. 10.1016/j.bbrc.2012.06.073 22728879

[DMM050361C8] Aruffo, A., Stamenkovic, I., Melnick, M., Underhill, C. B. and Seed, B. (1990). CD44 is the principal cell surface receptor for hyaluronate. *Cell* 61, 1303-1313. 10.1016/0092-8674(90)90694-a 1694723

[DMM050361C9] Atchison, L., Abutaleb, N. O., Snyder-Mounts, E., Gete, Y., Ladha, A., Ribar, T., Cao, K. and Truskey, G. A. (2020). iPSC-derived endothelial cells affect vascular function in a tissue-engineered blood vessel model of Hutchinson-Gilford progeria syndrome. *Stem Cell Rep.* 14, 325-337. 10.1016/j.stemcr.2020.01.005 PMC7013250 32032552

[DMM050361C10] Baeriswyl, D. C., Prionisti, I., Peach, T., Tsolkas, G., Chooi, K. Y., Vardakis, J., Morel, S., Diagbouga, M. R., Bijlenga, P., Cuhlmann, S. et al. (2019). Disturbed flow induces a sustained, stochastic NF-ΚB activation which may support intracranial aneurysm growth in vivo. *Sci. Rep.* 9, 4738. 10.1038/s41598-019-40959-y 30894565 PMC6426999

[DMM050361C11] Baeyens, N., Mulligan-Kehoe, M. J., Corti, F., Simon, D. D., Ross, T. D., Rhodes, J. M., Wang, T. Z., Mejean, C. O., Simons, M., Humphrey, J. et al. (2014). Syndecan 4 is required for endothelial alignment in flow and atheroprotective signaling. *Proc. Natl Acad. Sci. USA* 111, 17308-17313. 10.1073/pnas.1413725111 25404299 PMC4260558

[DMM050361C12] Baeyens, N., Nicoli, S., Coon, B. G., Ross, T. D., Van Den Dries, K., Han, J., Lauridsen, H. M., Mejean, C. O., Eichmann, A., Thomas, J.-L. et al. (2015). Vascular remodeling is governed by a VEGFR3-dependent fluid shear stress set point. *ELife* 4, e04645. 10.7554/eLife.04645 25643397 PMC4337723

[DMM050361C13] Baker, P. B., Baba, N. and Boesel, C. P. (1981). Cardiovascular abnormalities in progeria. case report and review of the literature. *Arch. Pathol. Lab. Med.* 105, 384-386. http://europepmc.org/abstract/MED/6894691. 6894691

[DMM050361C14] Bischoff, J. (2019). Endothelial-to-mesenchymal transition. *Circ. Res.* 124, 1163-1165. 10.1161/CIRCRESAHA.119.314813 30973806 PMC6540806

[DMM050361C15] Bondareva, O., Tsaryk, R., Bojovic, V., Odenthal-Schnittler, M., Siekmann, A. F. and Schnittler, H.-J. (2019). Identification of atheroprone shear stress responsive regulatory elements in endothelial cells. *Cardiovasc. Res.* 115, 1487-1499. 10.1093/cvr/cvz027 30785199

[DMM050361C16] Bosseboeuf, E., Chikh, A., Chaker, A. B., Mitchell, T. P., Vignaraja, D., Rajendrakumar, R., Khambata, R. S., Nightingale, T. D., Mason, J. C., Randi, A. M. et al. (2023). Neuropilin-1 interacts with VE-cadherin and TGFBR2 to stabilize adherens junctions and prevent activation of endothelium under flow. *Sci. Signal.* 16, eabo4863. 10.1126/scisignal.abo4863 37220183 PMC7614756

[DMM050361C17] Broadhouse, K. M., Price, A. N., Finnemore, A. E., Cox, D. J., David Edwards, A., Hajnal, J. V. and Groves, A. M. (2015). 4D phase contrast MRI in the preterm infant: visualisation of patent ductus arteriosus. *Arch. Dis. Child. Fetal Neonatal Ed.* 100, F164. 10.1136/archdischild-2013-305281 24907162

[DMM050361C18] Buglak, D. B., Bougaran, P., Kulikauskas, M. R., Liu, Z., Monaghan-Benson, E., Gold, A. L., Marvin, A. P., Burciu, A., Tanke, N. T., Oatley, M. et al. (2023). Nuclear SUN1 stabilizes endothelial cell junctions via microtubules to regulate blood vessel formation. *ELife* 12, e83652. 10.7554/eLife.83652 36989130 PMC10059686

[DMM050361C19] Cai, M., Huang, Y., Ghirlando, R., Wilson, K. L., Craigie, R. and Clore, G. M. (2001). Solution structure of the constant region of nuclear envelope protein LAP2 reveals two LEM-domain structures: one binds BAF and the other binds DNA. *EMBO J.* 20, 4399-4407. 10.1093/emboj/20.16.4399 11500367 PMC125263

[DMM050361C20] Chachisvilis, M., Zhang, Y.-L. and Frangos, J. A. (2006). G Protein-coupled receptors sense fluid shear stress in endothelial cells. *Proc. Natl Acad. Sci. USA* 103, 15463-15468. 10.1073/pnas.0607224103 17030791 PMC1622845

[DMM050361C21] Chambliss, A. B., Khatau, S. B., Erdenberger, N., Kyle Robinson, D., Hodzic, D., Longmore, G. D. and Wirtz, D. (2013). The LINC-anchored actin cap connects the extracellular milieu to the nucleus for ultrafast mechanotransduction. *Sci. Rep.* 3, 1087. 10.1038/srep01087 23336069 PMC3548190

[DMM050361C22] Chancellor, T. J., Lee, J., Thodeti, C. K. and Lele, T. (2010). Actomyosin tension exerted on the nucleus through nesprin-1 connections influences endothelial cell adhesion, migration, and cyclic strain-induced reorientation. *Biophys. J.* 99, 115-123. 10.1016/j.bpj.2010.04.011 20655839 PMC2895377

[DMM050361C23] Chang, L., Azzolin, L., Di Biagio, D., Zanconato, F., Battilana, G., Xiccato, R. L., Aragona, M., Giulitti, S., Panciera, T., Gandin, A. et al. (2018). The SWI/SNF complex is a mechanoregulated inhibitor of YAP and TAZ. *Nature* 563, 265-269. 10.1038/s41586-018-0658-1 30401838 PMC7612964

[DMM050361C24] Chen, P.-Y., Schwartz, M. A. and Simons, M. (2020). Endothelial-to-mesenchymal transition, vascular inflammation, and atherosclerosis. *Front. Cardiovasc. Med.* 7, 53. 10.3389/fcvm.2020.00053 32478094 PMC7232582

[DMM050361C25] Cheng, J., Koenig, S. N., Kuivaniemi, H. S., Garg, V. and Hans, C. P. (2023). Pharmacological inhibitor of notch signaling stabilizes the progression of small abdominal aortic aneurysm in a mouse model. *J. Am. Heart Assoc.* 3, e001064. 10.1161/JAHA.114.001064 PMC4338693 25349182

[DMM050361C26] Chien, S. (2008). Effects of disturbed flow on endothelial cells. *Ann. Biomed. Eng.* 36, 554-562. 10.1007/s10439-007-9426-3 18172767 PMC3718045

[DMM050361C27] Chou, E., Pirruccello, J. P., Ellinor, P. T. and Lindsay, M. E. (2023). Genetics and mechanisms of thoracic aortic disease. *Nat. Rev. Cardiol.* 20, 168-180. 10.1038/s41569-022-00763-0 36131050 PMC12175203

[DMM050361C28] Champy, C. and Carleton, H. M. (1921). Memoirs: observations on the shape of the nucleus and its determination. *J. Cell Sci.* s2-65, 589-625. 10.1242/jcs.s2-65.260.589

[DMM050361C29] Chuaiphichai, S., Rashbrook, V. S., Hale, A. B., Trelfa, L., Patel, J., Mcneill, E., Lygate, C. A., Channon, K. M. and Douglas, G. (2018). Endothelial cell tetrahydrobiopterin modulates sensitivity to Ang (Angiotensin) II-induced vascular remodeling, blood pressure, and abdominal aortic aneurysm. *Hypertension* 72, 128-138. 10.1161/HYPERTENSIONAHA.118.11144 29844152 PMC6012043

[DMM050361C30] Chuntharpursat-Bon, E., Povstyan, O. V., Ludlow, M. J., Carrier, D. J., Debant, M., Shi, J., Gaunt, H. J., Bauer, C. C., Curd, A., Simon Futers, T. et al. (2023). PIEZO1 and PECAM1 interact at cell-cell junctions and partner in endothelial force sensing. *Commun. Biol.* 6, 358. 10.1038/s42003-023-04706-4 37005489 PMC10067937

[DMM050361C31] Coleman, P. R., Lay, A. J., Ting, K. K., Zhao, Y., Li, J., Jarrah, S., Vadas, M. A. and Gamble, J. R. (2020). YAP and the RhoC regulator ARHGAP18, are required to mediate flow-dependent endothelial cell alignment. *Cell Commun. Signal.* 18, 18. 10.1186/s12964-020-0511-7 32013974 PMC6998144

[DMM050361C32] Conway, D. E., Breckenridge, M. T., Hinde, E., Gratton, E., Chen, C. S. and Schwartz, M. A. (2013). Fluid Shear Stress on Endothelial Cells Modulates Mechanical Tension across VE-Cadherin and PECAM-1. *Curr. Biol.* 23, 1024-1030. 10.1016/j.cub.2013.04.049 23684974 PMC3676707

[DMM050361C33] Coon, B. G., Baeyens, N., Han, J., Budatha, M., Ross, T. D., Fang, J. S., Yun, S., Thomas, J.-L. and Schwartz, M. A. (2015). Intramembrane binding of VE-cadherin to VEGFR2 and VEGFR3 assembles the endothelial mechanosensory complex. *J. Cell Biol.* 208, 975-986. 10.1083/jcb.201408103 25800053 PMC4384728

[DMM050361C34] Coon, B. G., Timalsina, S., Astone, M., Zhuang, Z. W., Fang, J., Han, J., Themen, J., Chung, M., Yang-Klingler, Y. J., Jain, M. et al. (2022). A mitochondrial contribution to anti-inflammatory shear stress signaling in vascular endothelial cells. *J. Cell Biol.* 221, e202109144. 10.1083/jcb.202109144 35695893 PMC9198948

[DMM050361C35] Chee, L. M., Frederic, B. B., Griffith, B. G. C., Loftus, A. E. P., Kumar, Y., Wills, J. C., Lee, M., Valli, J., Wheeler, A. P., Armstrong, J. D. et al. (2023). Mena regulates nesprin-2 to control actin–nuclear lamina associations, trans-nuclear membrane signalling and gene expression. *Nat. Commun.* 14, 1602. 10.1038/s41467-023-37021-x 36959177 PMC10036544

[DMM050361C36] Crisp, M., Liu, Q., Roux, K., Rattner, J. B., Shanahan, C., Burke, B., Stahl, P. D. and Hodzic, D. (2005). Coupling of the Nucleus and Cytoplasm: role of the LINC complex. *J. Cell Biol.* 172, 41-53. 10.1083/jcb.200509124 16380439 PMC2063530

[DMM050361C37] Dahl, K. N., Scaffidi, P., Islam, M. F., Yodh, A. G., Wilson, K. L. and Misteli, T. (2006). Distinct structural and mechanical properties of the nuclear lamina in Hutchinson–Gilford progeria syndrome. *Proc. Natl Acad. Sci. USA* 103, 10271-10276. 10.1073/pnas.0601058103 16801550 PMC1502447

[DMM050361C38] Dahl, K. N., Ribeiro, A. J. S. and Lammerding, J. (2008). Nuclear shape, mechanics, and mechanotransduction. *Circ. Res.* 102, 1307-1318. 10.1161/CIRCRESAHA.108.173989 18535268 PMC2717705

[DMM050361C39] Danielsson, B. E., Peters, H. C., Bathula, K., Spear, L. M., Noll, N. A., Dahl, K. N. and Conway, D. E. (2022a). Progerin-expressing endothelial cells are unable to adapt to shear stress. *Biophys. J.* 121, 620-628. 10.1016/j.bpj.2022.01.004 34999130 PMC8873939

[DMM050361C40] Danielsson, B. E., Tieu, K. V., Spagnol, S. T., Vu, K. K., Cabe, J. I., Raisch, T. B., Dahl, K. N. and Conway, D. E. (2022b). Chromatin condensation regulates endothelial cell adaptation to shear stress. *Mol. Biol. Cell* 33, ar101. 10.1091/mbc.E22-02-0064 35895088 PMC9582801

[DMM050361C41] Davies, P. F. (1995). Flow-mediated endothelial mechanotransduction. *Physiol. Rev.* 75, 519-560. 10.1152/physrev.1995.75.3.519 7624393 PMC3053532

[DMM050361C42] Davies, P. F., Remuzzi, A., Gordon, E. J., Dewey, C. F. and Gimbrone, M. A. (1986). Turbulent fluid shear stress induces vascular endothelial cell turnover in vitro. *Proc. Natl Acad. Sci. USA* 83, 2114-2117. 10.1073/pnas.83.7.2114 3457378 PMC323241

[DMM050361C43] Davies, P. F., Robotewskyj, A. and Griem, M. L. (1993). Endothelial cell adhesion in real time. measurements in vitro by tandem scanning confocal image analysis. *J. Clin. Invest.* 91, 2640-2652. 10.1172/JCI116503 8514872 PMC443328

[DMM050361C44] Davies, P. F., Robotewskyj, A. and Griem, M. L. (1994). Quantitative studies of endothelial cell adhesion. directional remodeling of focal adhesion sites in response to flow forces. *J. Clin. Invest.* 93, 2031-2038. 10.1172/JCI117197 8182135 PMC294317

[DMM050361C45] Davies, P. F., Manduchi, E., Stoeckert, C. J., Jiménez, J. M. and Jiang, Y.-Z. (2014). Emerging topic: flow-related epigenetic regulation of endothelial phenotype through DNA methylation. *Vascul. Pharmacol.* 62, 88-93. 10.1016/j.vph.2014.05.007 24874278 PMC4116435

[DMM050361C46] Deguchi, S., Maeda, K., Ohashi, T. and Sato, M. (2005). Flow-induced hardening of endothelial nucleus as an intracellular stress-bearing organelle. *J. Biomech.* 38, 1751-1759. 10.1016/j.jbiomech.2005.06.003 16005465

[DMM050361C47] Dekker, R. J., Van Soest, S., Fontijn, R. D., Salamanca, S., De Groot, P. G., Vanbavel, E., Pannekoek, H. and Horrevoets, A. J. G. (2002). Prolonged fluid shear stress induces a distinct set of endothelial cell genes, most specifically lung krüppel-like factor (KLF2). *Blood* 100, 1689-1698. 10.1182/blood-2002-01-0046 12176889

[DMM050361C48] Dekker, R. J., Van Thienen, J. V., Rohlena, J., De Jager, S. C., Elderkamp, Y. W., Seppen, J., De Vries, C. J. M., Biessen, E. A. L., Van Berkel, T. J. C., Pannekoek, H. et al. (2005). Endothelial KLF2 links local arterial shear stress levels to the expression of vascular tone-regulating genes. *Am. J. Pathol.* 167, 609-618. 10.1016/S0002-9440(10)63002-7 16049344 PMC1603569

[DMM050361C49] Denis, K. B., Cabe, J. I., Danielsson, B. E., Tieu, K. V., Mayer, C. R. and Conway, D. E. (2021). The LINC complex is required for endothelial cell adhesion and adaptation to shear stress and cyclic stretch. *Mol. Biol. Cell* 32, 1654-1663. 10.1091/mbc.E20-11-0698 34191529 PMC8684736

[DMM050361C50] De Sandre-Giovannoli, A. D., Bernard, R., Cau, P., Navarro, C., Amiel, J., Boccaccio, I., Lyonnet, S., Stewart, C. L., Munnich, A., Le Merrer, M. et al. (2003). Lamin a truncation in Hutchinson-Gilford progeria. *Science* 300, 2055. 10.1126/science.1084125 12702809

[DMM050361C51] Dewey, C. F., Jr, Bussolari, S. R., Gimbrone, M. A., Jr and Davies, P. F. (1981). The dynamic response of vascular endothelial cells to fluid shear stress. *J. Biomech. Eng.* 103, 177-185. 10.1115/1.3138276 7278196

[DMM050361C52] Dietz, H. C. and Pyeritz, R. E. (1995). Mutations in the human gene for fibrillin-1 (FBN1) in the Marfan syndrome and related disorders. *Hum. Mol. Genet.* 4 Suppl. 1, 1799-1809. 10.1093/hmg/4.suppl_1.1799 8541880

[DMM050361C53] Dinsmore, C. and Reiter, J. F. (2016). Endothelial primary cilia inhibit atherosclerosis. *EMBO Rep.* 17, 156-166. 10.15252/embr.201541019 26769565 PMC5290813

[DMM050361C54] Douglas, G., Mehta, V., Al Haj Zen, A., Akoumianakis, I., Goel, A., Rashbrook, V. S., Trelfa, L., Donovan, L., Drydale, E., Chuaiphichai, S. et al. (2020). A key role for the novel coronary artery disease gene JCAD in atherosclerosis via shear stress mechanotransduction. *Cardiovasc. Res.* 116, 1863-1874. 10.1093/cvr/cvz263 31584065 PMC7449560

[DMM050361C55] Duchemin, A.-L., Vignes, H. and Vermot, J. (2019). Mechanically activated piezo channels modulate outflow tract valve development through the Yap1 and Klf2-notch signaling axis. *ELife* 8, e44706. 10.7554/eLife.44706 31524599 PMC6779468

[DMM050361C56] Dunn, J., Qiu, H., Kim, S., Jjingo, D., Hoffman, R., Kim, C. W., Jang, I., Son, D. J., Kim, D., Pan, C. et al. (2014). Flow-dependent epigenetic DNA methylation regulates endothelial gene expression and atherosclerosis. *J. Clin. Invest.* 124, 3187-3199. 10.1172/JCI74792 24865430 PMC4071393

[DMM050361C57] Dupont, S., Morsut, L., Aragona, M., Enzo, E., Giulitti, S., Cordenonsi, M., Zanconato, F., Le Digabel, J., Forcato, M., Bicciato, S. et al. (2011). Role of YAP/TAZ in mechanotransduction. *Nature* 474, 179-183. 10.1038/nature10137 21654799

[DMM050361C58] Engelbrecht, E., Levesque, M. V., He, L., Vanlandewijck, M., Nitzsche, A., Niazi, H., Kuo, A., Singh, S. A., Aikawa, M., Holton, K. et al. (2020). Sphingosine 1-phosphate-regulated transcriptomes in heterogenous arterial and lymphatic endothelium of the aorta. *ELife* 9, e52690. 10.7554/eLife.52690 32091396 PMC7054001

[DMM050361C59] Erdmann, J., Willenborg, C., Nahrstaedt, J., Preuss, M., König, I. R., Baumert, J., Linsel-Nitschke, P., Gieger, C., Tennstedt, S., Belcredi, P. et al. (2011). Genome-wide association study identifies a new locus for coronary artery disease on chromosome 10p11.23. *Eur. Heart J.* 32, 158-168. 10.1093/eurheartj/ehq405 21088011

[DMM050361C60] Erdogmus, S., Storch, U., Danner, L., Becker, J., Winter, M., Ziegler, N., Wirth, A., Offermanns, S., Hoffmann, C., Gudermann, T. et al. (2019). Helix 8 is the essential structural motif of mechanosensitive GPCRs. *Nat. Commun.* 10, 5784. 10.1038/s41467-019-13722-0 31857598 PMC6923424

[DMM050361C61] Flaherty, J. T., Pierce, J. E., Ferrans, V. J., Patel, D. J., Tucker, W. K. and Fry, D. L. (1972). Endothelial nuclear patterns in the canine arterial tree with particular reference to hemodynamic events. *Circ. Res.* 30, 23-33. 10.1161/01.RES.30.1.23 5007525

[DMM050361C62] Foisner, R. and Gerace, L. (1993). Integral membrane proteins of the nuclear envelope interact with lamins and chromosomes, and binding is modulated by mitotic phosphorylation. *Cell* 73, 1267-1279. 10.1016/0092-8674(93)90355-T 8324822

[DMM050361C63] Fong, L. G., Ng, J. K., Meta, M., Coté, N., Yang, S. H., Stewart, C. L., Sullivan, T., Burghardt, A., Majumdar, S., Reue, K. et al. (2004). Heterozygosity for lmna deficiency eliminates the progeria-like phenotypes in Zmpste24-deficient mice. *Proc. Natl Acad. Sci. USA* 101, 18111-18116. 10.1073/pnas.0408558102 15608054 PMC536056

[DMM050361C64] Fuglerud, B. M., Drissler, S., Lotto, J., Stephan, T. L., Thakur, A., Cullum, R. and Hoodless, P. A. (2022). SOX9 reprograms endothelial cells by altering the chromatin landscape. *Nucleic Acids Res.* 50, 8547-8565. 10.1093/nar/gkac652 35904801 PMC9410909

[DMM050361C65] Galvani, S., Sanson, M., Blaho, V. A., Swendeman, S. L., Obinata, H., Conger, H., Dahlbäck, B., Kono, M., Proia, R. L., Smith, J. D. et al. (2015). HDL-bound sphingosine 1-phosphate acts as a biased agonist for the endothelial cell receptor S1P1 to limit vascular inflammation. *Sci. Signal.* 8, ra79. 10.1126/scisignal.aaa2581 26268607 PMC4768813

[DMM050361C66] Goel, R., Schrank, B. R., Arora, S., Boylan, B., Fleming, B., Miura, H., Newman, P. J., Molthen, R. C. and Newman, D. K. (2008). Site-Specific Effects of PECAM-1 on atherosclerosis in LDL receptor–deficient mice. *Arterioscler. Thromb. Vasc. Biol.* 28, 1996-2002. 10.1161/ATVBAHA.108.172270 18669884 PMC3013511

[DMM050361C67] Goetz, J. G., Steed, E., Ferreira, R. R., Roth, S., Ramspacher, C., Boselli, F., Charvin, G., Liebling, M., Wyart, C., Schwab, Y. et al. (2014). Endothelial cilia mediate low flow sensing during zebrafish vascular development. *Cell Rep.* 6, 799-808. 10.1016/j.celrep.2014.01.032 24561257

[DMM050361C68] Goldman, R. D., Shumaker, D. K., Erdos, M. R., Eriksson, M., Goldman, A. E., Gordon, L. B., Gruenbaum, Y., Khuon, S., Mendez, M., Varga, R. et al. (2004). Accumulation of mutant lamin a causes progressive changes in nuclear architecture in Hutchinson–Gilford progeria syndrome. *Proc. Natl Acad. Sci. USA* 101, 8963-8968. 10.1073/pnas.0402943101 15184648 PMC428455

[DMM050361C69] Guilluy, C., Osborne, L. D., Van Landeghem, L., Sharek, L., Superfine, R., Garcia-Mata, R. and Burridge, K. (2014). Isolated nuclei adapt to force and reveal a mechanotransduction pathway in the nucleus. *Nat. Cell Biol.* 16, 376-381. 10.1038/ncb2927 24609268 PMC4085695

[DMM050361C70] Gupta, A., Thirugnanam, K., Thamilarasan, M., Mohieldin, A. M., Zedan, H. T., Prabhudesai, S., Griffin, M. R., Spearman, A. D., Pan, A., Palecek, S. P. et al. (2022). Cilia proteins are biomarkers of altered flow in the vasculature. *JCI Insight* 7, e151813. 10.1172/jci.insight.151813 35143420 PMC8986075

[DMM050361C71] Hahn, C. and Schwartz, M. A. (2009). Mechanotransduction in vascular physiology and atherogenesis. *Nat. Rev. Mol. Cell Biol.* 10, 53-62. 10.1038/nrm2596 19197332 PMC2719300

[DMM050361C72] Hajra, L., Evans, A. I., Chen, M., Hyduk, S. J., Collins, T. and Cybulsky, M. I. (2000). The NF-ΚB signal transduction pathway in aortic endothelial cells is primed for activation in regions predisposed to atherosclerotic lesion formation. *Proc. Natl Acad. Sci. USA* 97, 9052-9057. 10.1073/pnas.97.16.9052 10922059 PMC16820

[DMM050361C73] Hamik, A., Lin, Z., Kumar, A., Balcells, M., Sinha, S., Katz, J., Feinberg, M. W., Gerszten, R. E., Edelman, E. R. and Jain, M. K. (2007). Kruppel-like factor 4 regulates endothelial inflammation. *J. Biol. Chem.* 282, 13769-13779. 10.1074/jbc.M700078200 17339326

[DMM050361C74] Hans, C. P., Koenig, S. N., Huang, N., Cheng, J., Beceiro, S., Guggilam, A., Kuivaniemi, H., Partida-Sánchez, S. and Garg, V. (2012). Inhibition of notch1 signaling reduces abdominal aortic aneurysm in mice by attenuating macrophage-mediated inflammation. *Arterioscler. Thromb. Vasc. Biol.* 32, 3012-3023. 10.1161/ATVBAHA.112.254219 23087364 PMC4467024

[DMM050361C75] Haque, F., Lloyd, D. J., Smallwood, D. T., Dent, C. L., Shanahan, C. M., Fry, A. M., Trembath, R. C. and Shackleton, S. (2006). SUN1 interacts with nuclear lamin a and cytoplasmic nesprins to provide a physical connection between the nuclear lamina and the cytoskeleton. *Mol. Cell. Biol.* 26, 3738-3751. 10.1128/MCB.26.10.3738-3751.2006 16648470 PMC1488999

[DMM050361C76] Harding, I. C., Mitra, R., Mensah, S. A., Herman, I. M. and Ebong, E. E. (2018). Pro-atherosclerotic disturbed flow disrupts caveolin-1 expression, localization, and function via Glycocalyx degradation. *J. Transl. Med.* 16, 364. 10.1186/s12967-018-1721-2 30563532 PMC6299559

[DMM050361C77] Harloff, A., Nussbaumer, A., Bauer, S., Stalder, A. F., Frydrychowicz, A., Weiller, C., Hennig, J. and Markl, M. (2010). In Vivo Assessment of Wall shear stress in the atherosclerotic aorta using flow-sensitive 4D MRI. *Magn. Reson. Med.* 63, 1529-1536. 10.1002/mrm.22383 20512856

[DMM050361C78] He, M., Huang, T.-S., Li, S., Hong, H.-C., Chen, Z., Martin, M., Zhou, X., Huang, H.-Y., Su, S.-H., Zhang, J. et al. (2019). Atheroprotective flow upregulates ITPR3 (Inositol 1,4,5-Trisphosphate Receptor 3) in vascular endothelium via KLF4 (Krüppel-Like Factor 4)-mediated histone modifications. *Arterioscler. Thromb. Vasc. Biol.* 39, 902-914. 10.1161/ATVBAHA.118.312301 30917677 PMC6536300

[DMM050361C79] Hernandez, G. E., Ma, F., Martinez, G., Firozabadi, N. B., Salvador, J., Juang, L. J., Leung, J., Zhao, P., López, D. A., Ardehali, R. et al. (2022). Aortic intimal resident macrophages are essential for maintenance of the non-thrombogenic intravascular state. *Nat. Cardiovas. Res.* 1, 67-84. 10.1038/s44161-021-00006-4 PMC9121812 35599984

[DMM050361C80] Hsu, P.-L., Chen, J.-S., Wang, C.-Y., Wu, H.-L. and Mo, F.-E. (2019). Shear-induced CCN1 promotes atheroprone endothelial phenotypes and atherosclerosis. *Circulation* 139, 2877-2891. 10.1161/CIRCULATIONAHA.118.033895 30917686

[DMM050361C81] Hu, Y., Chen, M., Wang, M. and Li, X. (2022). Flow-mediated vasodilation through mechanosensitive G Protein-coupled receptors in endothelial cells. *Trends Cardiovasc. Med.* 32, 61-70. 10.1016/j.tcm.2020.12.010 33406458

[DMM050361C82] Iiyama, K., Hajra, L., Iiyama, M., Li, H., Dichiara, M., Medoff, B. D. and Cybulsky, M. I. (1999). Patterns of vascular cell adhesion molecule-1 and intercellular adhesion molecule-1 expression in rabbit and mouse atherosclerotic lesions and at sites predisposed to lesion formation. *Circ. Res.* 85, 199-207. 10.1161/01.res.85.2.199 10417402

[DMM050361C83] Illi, B., Nanni, S., Scopece, A., Farsetti, A., Biglioli, P., Capogrossi, M. C. and Gaetano, C. (2003). Shear stress–mediated chromatin remodeling provides molecular basis for flow-dependent regulation of gene expression. *Circ. Res.* 93, 155-161. 10.1161/01.RES.0000080933.82105.29 12805238

[DMM050361C84] Iring, A., Jin, Y.-J., Albarrán-Juárez, J., Siragusa, M., Wang, S., Dancs, P. T., Nakayama, A., Tonack, S., Chen, M., Künne, C. et al. (2019). Shear stress-induced endothelial adrenomedullin signaling regulates vascular tone and blood pressure. *J. Clin. Invest.* 129, 2775-2791. 10.1172/JCI123825 31205027 PMC6597232

[DMM050361C153] Ishibashi, S., Brown, M. S., Goldstein, J. L., Gerard, R. D., Hammer, R. E. and Herz, J. (1993). Hypercholesterolemia in low density lipoprotein receptor knockout mice and its reversal by adenovirus-mediated gene delivery. *J Clin Invest.* 92, 883-893. 10.1172/JCI116663 8349823 PMC294927

[DMM050361C85] Jia, M., Li, Q., Guo, J., Shi, W., Zhu, L., Huang, Y., Li, Y., Wang, L., Ma, S., Zhuang, T. et al. (2022). Deletion of BACH1 Attenuates Atherosclerosis by Reducing Endothelial Inflammation. *Circ. Res.* 130, 1038-1055. 10.1161/CIRCRESAHA.121.319540 35196865

[DMM050361C86] Jiang, Y.-Z., Jiménez, J. M., Ou, K., Mccormick, M. E., Zhang, L.-D. and Davies, P. F. (2014). Hemodynamic disturbed flow induces differential DNA methylation of endothelial kruppel-like factor 4 promoter in vitro and in vivo. *Circ. Res.* 115, 32-43. 10.1161/CIRCRESAHA.115.303883 24755985 PMC4065854

[DMM050361C87] Jiang, W., Agrawal, D. K. and Boosani, C. S. (2018). Cell-specific histone modifications in atherosclerosis (Review). *Mol. Med. Rep.* 18, 1215-1224. 10.3892/mmr.2018.9142 29901135 PMC6072136

[DMM050361C88] Jung, B., Obinata, H., Galvani, S., Mendelson, K., Ding, B.-., Skoura, A., Kinzel, B., Brinkmann, V., Rafii, S., Evans, T. et al. (2012). Flow-regulated endothelial S1P receptor-1 signaling sustains vascular development. *Dev. Cell* 23, 600-610. 10.1016/j.devcel.2012.07.015 22975328 PMC3443394

[DMM050361C89] Kalluri, A. S., Vellarikkal, S. K., Edelman, E. R., Nguyen, L., Subramanian, A., Ellinor, P. T., Regev, A., Kathiresan, S. and Gupta, R. M. (2019). Single-cell analysis of the normal mouse aorta reveals functionally distinct endothelial cell populations. *Circulation* 140, 147-163. 10.1161/CIRCULATIONAHA.118.038362 31146585 PMC6693656

[DMM050361C90] Khachigian, L. M., Resnick, N., Gimbrone, M. A., Jr and Collins, T. (1995). Nuclear factor-kappa b interacts functionally with the platelet-derived growth factor b-chain shear-stress response element in vascular endothelial cells exposed to fluid shear stress. *J. Clin. Invest.* 96, 1169-1175. 10.1172/JCI118106 7635955 PMC185309

[DMM050361C91] King, S. J., Nowak, K., Suryavanshi, N., Holt, I., Shanahan, C. M. and Ridley, A. J. (2014). Nesprin-1 and nesprin-2 regulate endothelial cell shape and migration. *Cytoskeleton* 71, 423-434. 10.1002/cm.21182 24931616

[DMM050361C92] Lee, D.-Y., Lee, C.-I., Lin, T.-E., Lim, S. H., Zhou, J., Tseng, Y.-C., Chien, S. and Chiu, J.-J. (2012). Role of histone deacetylases in transcription factor regulation and cell cycle modulation in endothelial cells in response to disturbed flow. *Proc. Natl Acad. Sci. USA* 109, 1967-1972. 10.1073/pnas.1121214109 22308472 PMC3277521

[DMM050361C93] Levitt, M. R., Mandrycky, C., Abel, A., Kelly, C. M., Levy, S., Chivukula, V. K., Zheng, Y., Aliseda, A. and Kim, L. J. (2019). Genetic correlates of wall shear stress in a patient-Specific 3D-printed cerebral aneurysm model. *J. Neurointerv. Surg.* 11, 999-1003. 10.1136/neurintsurg-2018-014669 30979845 PMC6744304

[DMM050361C94] Li, J., Hou, B., Tumova, S., Muraki, K., Bruns, A., Ludlow, M. J., Sedo, A., Hyman, A. J., Mckeown, L., Young, R. S. et al. (2014). Piezo1 integration of vascular architecture with physiological force. *Nature* 515, 279-282. 10.1038/nature13701 25119035 PMC4230887

[DMM050361C95] Li, B., He, J., Lv, H., Liu, Y., Lv, X., Zhang, C., Zhu, Y. and Ai, D. (2019). C-Abl regulates YAPY357 phosphorylation to activate endothelial atherogenic responses to disturbed flow. *J. Clin. Invest.* 129, 1167-1179. 10.1172/JCI122440 30629551 PMC6391101

[DMM050361C96] Liu, Y., Sweet, D. T., Irani-Tehrani, M., Maeda, N. and Tzima, E. (2008). Shc coordinates signals from intercellular junctions and integrins to regulate flow-induced inflammation. *J. Cell Biol.* 182, 185-196. 10.1083/jcb.200709176 18606845 PMC2447891

[DMM050361C97] Liu, C., Shen, M., Tan, W. L. W., Chen, I. Y., Liu, Y., Yu, X., Yang, H., Zhang, A., Liu, Y., Zhao, M.-T. et al. (2023). Statins improve endothelial function via suppression of epigenetic-driven EndMT. *Nat. Cardiovasc. Res.* 2, 467-485. 10.1038/s44161-023-00267-1 37693816 PMC10489108

[DMM050361C98] Lowis, C., Winaya, A. R., Kumari, P., Rivera, C. F., Vlahos, J., Hermantara, R., Pratama, M. Y. and Ramkhelawon, B. (2023). Mechanosignals in abdominal aortic aneurysms. *Front. Cardiovas. Med*. 9, 1021934. 10.3389/fcvm.2022.1021934 PMC9868277 36698932

[DMM050361C152] Lu, Y. W., Martino, N., Gerlach, B. D., Lamar, J. M., Vincent, P. A., Adam, A. P. and Schwarz, J. J. (2021). MEF2 (Myocyte Enhancer Factor 2) Is Essential for Endothelial Homeostasis and the Atheroprotective Gene Expression Program. *Arterioscler Thromb Vasc Biol*. 41, 1105-1123. 10.1161/ATVBAHA.120.314978 33406884 PMC7938420

[DMM050361C99] Luu, V. Z., Chowdhury, B., Al-Omran, M., Hess, D. A. and Verma, S. (2018). Role of endothelial primary cilia as fluid mechanosensors on vascular health. *Atherosclerosis* 275, 196-204. 10.1016/j.atherosclerosis.2018.06.818 29945035

[DMM050361C100] Mack, J. J., Mosqueiro, T. S., Archer, B. J., Jones, W. M., Sunshine, H., Faas, G. C., Briot, A., Aragón, R. L., Su, T., Romay, M. C. et al. (2017). NOTCH1 is a mechanosensor in adult arteries. *Nat. Commun.* 8, 1620. 10.1038/s41467-017-01741-8 29158473 PMC5696341

[DMM050361C101] Mahmoud, M., Souilhol, C., Serbanovic-Canic, J. and Evans, P. (2019). GATA4-Twist1 signalling in disturbed flow-induced atherosclerosis. *Cardiovasc. Drugs Ther.* 33, 231-237. 10.1007/s10557-019-06863-3 30809744

[DMM050361C102] Manilal, S., Nguyen, T. M., Sewry, C. A. and Morris, G. E. (1996). The emery-dreifuss muscular dystrophy protein, emerin, is a nuclear membrane protein. *Hum. Mol. Genet.* 5, 801-808. 10.1093/hmg/5.6.801 8776595

[DMM050361C103] Maniotis, A., Chen, C. and Ingber, D. (1997). Demonstration of mechanical connections between integrins, cytoskeletal filaments, and nucleoplasm that stabilize nuclear structure. *Proc. Natl Acad. Sci. USA* 94, 849-854. 10.1073/pnas.94.3.849 9023345 PMC19602

[DMM050361C104] Mannion, A. J., Zhao, H., Zhang, Y., Von Wright, Y., Bergman, O., Saharinen, P. and Holmgren, L. (2023). The junctional mechanosensor AmotL2 regulates YAP promotor accessibility. *bioRxiv* 2023.01.13.523596. 10.1101/2023.01.13.523596

[DMM050361C105] Martinac, B. (2004). Mechanosensitive ion channels: molecules of mechanotransduction. *J. Cell Sci.* 117, 2449-2460. 10.1242/jcs.01232 15159450

[DMM050361C106] Mccormick, S. M., Eskin, S. G., Mcintire, L. V., Teng, C. L., Lu, C. M., Russell, C. G. and Chittur, K. K. (2001). DNA microarray reveals changes in gene expression of shear stressed human umbilical vein endothelial cells. *Proc. Natl. Acad. Sci. U.S.A.* 98, 8955-8960. 10.1073/pnas.171259298 11481467 PMC55355

[DMM050361C107] Mehta, V., Pang, K.-L., Rozbesky, D., Nather, K., Keen, A., Lachowski, D., Kong, Y., Karia, D., Ameismeier, M., Huang, J. et al. (2020). The guidance receptor plexin D1 is a mechanosensor in endothelial cells. *Nature* 578, 290-295. 10.1038/s41586-020-1979-4 32025034 PMC7025890

[DMM050361C108] Mehta, V., Pang, K.-L., Givens, C. S., Chen, Z., Huang, J., Sweet, D. T., Jo, H., Reader, J. S. and Tzima, E. (2023). Mechanical forces regulate endothelial-to-mesenchymal transition and atherosclerosis via an Alk5-Shc mechanotransduction pathway. *Sci. Adv.* 7, eabg5060. 10.1126/sciadv.abg5060 PMC8270486 34244146

[DMM050361C109] Mieremet, A., Van Der Stoel, M., Li, S., Coskun, E., Van Krimpen, T., Huveneers, S. and De Waard, V. (2022). Endothelial dysfunction in Marfan syndrome mice is restored by resveratrol. *Sci. Rep.* 12, 22504. 10.1038/s41598-022-26662-5 36577770 PMC9797556

[DMM050361C110] Miroshnikova, Y. A., Nava, M. M. and Wickström, S. A. (2017). Emerging roles of mechanical forces in chromatin regulation. *J. Cell Sci.* 130, 2243-2250. 10.1242/jcs.202192 28646093

[DMM050361C111] Mochizuki, S., Vink, H., Hiramatsu, O., Kajita, T., Shigeto, F., Spaan, J. A. E. and Kajiya, F. (2003). Role of hyaluronic acid glycosaminoglycans in shear-induced endothelium-derived nitric oxide release. *Am. J. Physiol. Heart Circ. Physiol.* 285, H722-H726. 10.1152/ajpheart.00691.2002 12730059

[DMM050361C112] Moonen, J.-R., Chappell, J., Shi, M., Shinohara, T., Li, D., Mumbach, M. R., Zhang, F., Nair, R. V., Nasser, J., Mai, D. H. et al. (2022). KLF4 recruits SWI/SNF to increase chromatin accessibility and reprogram the endothelial enhancer landscape under laminar shear stress. *Nat. Commun.* 13, 4941. 10.1038/s41467-022-32566-9 35999210 PMC9399231

[DMM050361C113] Morgan, J. T., Pfeiffer, E. R., Thirkill, T. L., Kumar, P., Peng, G., Fridolfsson, H. N., Douglas, G. C., Starr, D. A. and Barakat, A. I. (2011). Nesprin-3 regulates endothelial cell morphology, perinuclear cytoskeletal architecture, and flow-induced polarization. *Mol. Biol. Cell* 22, 4324-4334. 10.1091/mbc.e11-04-0287 21937718 PMC3216658

[DMM050361C114] Mylvaganam, S., Plumb, J., Yusuf, B., Li, R., Lu, C.-Y., Robinson, L. A., Freeman, S. A. and Grinstein, S. (2022). The spectrin cytoskeleton integrates endothelial mechanoresponses. *Nat. Cell Biol.* 24, 1226-1238. 10.1038/s41556-022-00953-5 35817960

[DMM050361C115] Nagano, A., Koga, R., Ogawa, M., Kurano, Y., Kawada, J., Okada, R., Hayashi, Y. K., Tsukahara, T. and Arahata, K. (1996). Emerin deficiency at the nuclear membrane in patients with emery-dreifuss muscular dystrophy. *Nat. Genet.* 12, 254-259. 10.1038/ng0396-254 8589715

[DMM050361C154] Nagel, T., Resnick, N., Atkinson, W. J. and Dewey, C. F. Jr and Gimbrone, M. A. Jr. (1994). Shear stress selectively upregulates intercellular adhesion molecule-1 expression in cultured human vascular endothelial cells. *J Clin Invest.* 94, 885-891. 10.1172/JCI117410 7518844 PMC296171

[DMM050361C116] Nakajima, H., Yamamoto, K., Agarwala, S., Terai, K., Fukui, H., Fukuhara, S., Ando, K., Miyazaki, T., Yokota, Y., Schmelzer, E. et al. (2017). Flow-dependent endothelial YAP regulation contributes to vessel maintenance. *Dev. Cell* 40, 523-536.e6. 10.1016/j.devcel.2017.02.019 28350986

[DMM050361C117] Nakayama, A., Albarrán-Juárez, J., Liang, G., Roquid, K. A., Iring, A., Tonack, S., Chen, M., Müller, O. J., Weinstein, L. S. and Offermanns, S. (2020). Disturbed Flow-induced Gs-mediated signaling protects against endothelial inflammation and atherosclerosis. *JCI Insight* 5, e140485. 10.1172/jci.insight.140485 33268595 PMC7714404

[DMM050361C118] Nauli, S. M., Kawanabe, Y., Kaminski, J. J., Pearce, W. J., Ingber, D. E. and Zhou, J. (2008). Endothelial cilia are fluid shear sensors that regulate calcium signaling and nitric oxide production through polycystin-1. *Circulation* 117, 1161-1171. 10.1161/CIRCULATIONAHA.107.710111 18285569 PMC3071982

[DMM050361C119] Nava, M. M., Miroshnikova, Y. A., Biggs, L. C., Whitefield, D. B., Metge, F., Boucas, J., Vihinen, H., Jokitalo, E., Li, X., García Arcos, J. M. et al. (2020). Heterochromatin-driven nuclear softening protects the genome against mechanical stress-induced damage. *Cell* 181, 800-817.e22. 10.1016/j.cell.2020.03.052 32302590 PMC7237863

[DMM050361C155] Obaze, D. E. and Wright, H. P. (1968). A modified technique for producing “en face“ (Häutchen) preparations of endothelium for autoradiography. *J Atheroscler Res*. 8, 861-863. 10.1016/s0368-1319(68)80050-x 5688384

[DMM050361C120] Oechtering, T. H., Sieren, M. M., Hunold, P., Hennemuth, A., Huellebrand, M., Scharfschwerdt, M., Richardt, D., Sievers, H.-H., Barkhausen, J. and Frydrychowicz, A. (2020). Time-resolved 3-dimensional magnetic resonance phase contrast imaging (4D Flow MRI) reveals Altered blood flow patterns in the ascending aorta of patients with valve-sparing aortic root replacement. *J. Thorac. Cardiovasc. Surg.* 159, 798-810.e1. 10.1016/j.jtcvs.2019.02.127 31078313

[DMM050361C156] Ohtsuka, A., Ando, J., Korenaga, R., Kamiya, A., Toyama-Sorimachi, N. and Miyasaka, M. (1993). The effect of flow on the expression of vascular adhesion molecule-1 by cultured mouse endothelial cells. *Biochem. Biophys. Res. Commun.* 193, 303-310. 10.1006/bbrc.1993.1624 7684904

[DMM050361C121] Olive, M., Harten, I., Mitchell, R., Beers, J. K., Djabali, K., Cao, K., Erdos, M. R., Blair, C., Funke, B., Smoot, L. et al. (2010). Cardiovascular pathology in Hutchinson-Gilford progeria: correlation with the vascular pathology of aging. *Arterioscler. Thromb. Vasc. Biol.* 30, 2301-2309. 10.1161/ATVBAHA.110.209460 20798379 PMC2965471

[DMM050361C122] Osmanagic-Myers, S., Kiss, A., Manakanatas, C., Hamza, O., Sedlmayer, F., Szabo, P. L., Fischer, I., Fichtinger, P., Podesser, B. K., Eriksson, M. et al. (2019). Endothelial progerin expression causes cardiovascular pathology through an impaired mechanoresponse. *J. Clin. Invest.* 129, 531-545. 10.1172/JCI121297 30422822 PMC6355303

[DMM050361C123] Pahakis, M. Y., Kosky, J. R., Dull, R. O. and Tarbell, J. M. (2007). The role of endothelial glycocalyx components in mechanotransduction of fluid shear stress. *Biochem. Biophys. Res. Commun.* 355, 228-233. 10.1016/j.bbrc.2007.01.137 17291452 PMC1847369

[DMM050361C124] Parmar, K. M., Benjamin Larman, H., Dai, G., Zhang, Y., Wang, E. T., Moorthy, S. N. and Kratz, J. R. (2006). Integration of flow-dependent endothelial phenotypes by kruppel-like factor 2. *J. Clin. Invest.* 116, 49-58. 10.1172/JCI24787 16341264 PMC1307560

[DMM050361C125] Pereira, L., Andrikopoulos, K., Tian, J., Lee, S. Y., Keene, D. R., Ono, R., Reinhardt, D. P., Sakai, L. Y., Biery, N. J., Bunton, T. et al. (1997). Targetting of the gene encoding fibrillin–1 recapitulates the vascular aspect of Marfan syndrome. *Nat. Genet.* 17, 218-222. 10.1038/ng1097-218 9326947

[DMM050361C126] Rateri, D. L., Moorleghen, J. J., Balakrishnan, A., Phillip Owens, A., Howatt, D. A., Subramanian, V., Poduri, A., Charnigo, R., Cassis, L. A. and Daugherty, A. (2011). Endothelial cell–specific deficiency of Ang II type 1a receptors attenuates Ang II–induced ascending aortic aneurysms in LDL receptor−/− mice. *Circ. Res.* 108, 574-581. 10.1161/CIRCRESAHA.110.222844 21252156 PMC3076204

[DMM050361C127] Richter, R. P., Ashtekar, A. R., Zheng, L., Pretorius, D., Kaushlendra, T., Sanderson, R. D., Gaggar, A. and Richter, J. R. (2022). Glycocalyx heparan sulfate cleavage promotes endothelial cell angiopoietin-2 expression by impairing shear stress-related AMPK/FoxO1 signaling. *JCI Insight* 7, 15. 10.1172/jci.insight.155010 PMC9462499 35763350

[DMM050361C128] Salvador, J., Hernandez, G. E., Ma, F., Abrahamson, C. W., Pellegrini, M., Goldman, R., Ridge, K. M. and Luisa Iruela-Arispe, M. (2022). Transcriptional evaluation of the ductus arteriosus at the single-cell level uncovers a requirement for vim (Vimentin) for complete closure. *Arterioscler. Thromb. Vasc. Biol.* 42, 732-742. 10.1161/ATVBAHA.121.317172 35443793 PMC9806842

[DMM050361C129] Schneider, D. J. and Moore, J. W. (2006). Patent ductus arteriosus. *Circulation* 114, 1873-1882. 10.1161/CIRCULATIONAHA.105.592063 17060397

[DMM050361C130] Senbanerjee, S., Lin, Z., Brandon Atkins, G., Greif, D. M., Rao, R. M., Kumar, A., Feinberg, M. W., Chen, Z., Simon, D. I., Luscinskas, F. W. et al. (2004). KLF2 is a novel transcriptional regulator of endothelial proinflammatory activation. *J. Exp. Med.* 199, 1305-1315. 10.1084/jem.20031132 15136591 PMC2211816

[DMM050361C131] Silkworth, J. B., Stehbens, W. E. and Phil, D. (1975). The shape of endothelial cells in en face preparations of rabbit blood vessels. *Angiology* 26, 474-487. 10.1177/000331977502600607

[DMM050361C132] Sladitschek-Martens, H. L., Guarnieri, A., Brumana, G., Zanconato, F., Battilana, G., Xiccato, R. L., Panciera, T., Forcato, M., Bicciato, S., Guzzardo, V. et al. (2022). YAP/TAZ activity in stromal cells prevents ageing by controlling CGAS–STING. *Nature* 607, 790-798. 10.1038/s41586-022-04924-6 35768505 PMC7613988

[DMM050361C133] Souilhol, C., Harmsen, M. C., Evans, P. C. and Krenning, G. (2018). Endothelial–mesenchymal transition in atherosclerosis. *Cardiovasc. Res.* 114, 565-577. 10.1093/cvr/cvx253 29309526

[DMM050361C134] Souilhol, C., Ayllon, B. T., Li, X., Diagbouga, M. R., Zhou, Z., Canham, L., Roddie, H., Pirri, D., Chambers, E. V., Dunning, M. J. et al. (2023). JAG1-NOTCH4 mechanosensing drives atherosclerosis. *Sci. Adv.* 8, eabo7958. 10.1126/sciadv.abo7958 PMC9432841 36044575

[DMM050361C135] Sun, S., Qin, W., Tang, X., Meng, Y., Hu, W., Zhang, S., Qian, M., Liu, Z., Cao, X., Pang, Q. et al. (2023). Vascular endothelium–targeted Sirt7 gene therapy rejuvenates blood vessels and extends life span in a Hutchinson-Gilford progeria model. *Sci. Adv.* 6, eaay5556. 10.1126/sciadv.aay5556 PMC7030934 32128409

[DMM050361C136] Sweet, D. T., Chen, Z., Givens, C. S., Phillip Owens, A., Rojas, M. and Tzima, E. (2013). Endothelial Shc regulates arteriogenesis through dual control of arterial specification and inflammation via the notch and nuclear factor-κ–light-chain-enhancer of activated B-cell pathways. *Circ. Res.* 113, 32-39. 10.1161/CIRCRESAHA.113.301407 23661718 PMC3918667

[DMM050361C137] Swift, J., Ivanovska, I. L., Buxboim, A., Harada, T., Dingal, P. C. D. P., Pinter, J., Pajerowski, J. D., Spinler, K. R., Shin, J.-W., Tewari, M. et al. (2013). Nuclear lamin-A scales with tissue stiffness and enhances matrix-directed differentiation. *Science* 341, 1240104. 10.1126/science.1240104 23990565 PMC3976548

[DMM050361C138] Tamargo, I. A., Baek, K. I., Kim, Y., Park, C. and Jo, H. (2023). Flow-induced reprogramming of endothelial cells in atherosclerosis. *Nat. Rev. Cardiol.* 20, 738-753. 10.1038/s41569-023-00883-1 37225873 PMC10206587

[DMM050361C139] Tkachenko, E., Gutierrez, E., Saikin, S. K., Fogelstrand, P., Kim, C., Groisman, A. and Ginsberg, M. H. (2013). The nucleus of endothelial cell as a sensor of blood flow direction. *Biol. Open* 2, 1007-1012. 10.1242/bio.20134622 24167710 PMC3798183

[DMM050361C140] Tsaryk, R., Yucel, N., Leonard, E. V., Diaz, N., Bondareva, O., Odenthal-Schnittler, M., Arany, Z., Vaquerizas, J. M., Schnittler, H. and Siekmann, A. F. (2022). Shear stress switches the association of endothelial enhancers from ETV/ETS to KLF transcription factor binding sites. *Sci. Rep.* 12, 4795. 10.1038/s41598-022-08645-8 35314737 PMC8938417

[DMM050361C141] Tzima, E., Del Pozo, M. A., Kiosses, W. B., Mohamed, S. A., Li, S., Chien, S. and Schwartz, M. A. (2002). Activation of Rac1 by shear stress in endothelial cells mediates both cytoskeletal reorganization and effects on gene expression. *EMBO J.* 21, 6791-6800. 10.1093/emboj/cdf688 12486000 PMC139108

[DMM050361C157] Tzima, E., del Pozo, M. A., Shattil, S. J., Chien, S. and Schwartz, M. A. (2001). Activation of integrins in endothelial cells by fluid shear stress mediates Rho-dependent cytoskeletal alignment. *EMBO J.* 20, 4639-4647. 10.1093/emboj/20.17.4639 11532928 PMC125600

[DMM050361C142] Tzima, E., Irani-Tehrani, M., Kiosses, W. B., Dejana, E., Schultz, D. A., Engelhardt, B., Cao, G., Delisser, H. and Schwartz, M. A. (2005). A mechanosensory complex that mediates the endothelial cell response to fluid shear stress. *Nature* 437, 426-431. 10.1038/nature03952 16163360

[DMM050361C143] Van der Heiden, K., Hierck, B. P., Krams, R., De Crom, R., Cheng, C., Baiker, M., Pourquie, M. J. B. M., Alkemade, F. E., Deruiter, M. C., Gittenberger-De Groot, A. C. et al. (2008). Endothelial primary cilia in areas of disturbed flow are at the base of atherosclerosis. *Atherosclerosis* 196, 542-550. 10.1016/j.atherosclerosis.2007.05.030 17631294

[DMM050361C144] Wang, W., Ha, C. H., Jhun, B. S., Wong, C., Jain, M. K. and Jin, Z.-G. (2010). Fluid shear stress stimulates phosphorylation-dependent nuclear export of HDAC5 and mediates expression of KLF2 and ENOS. *Blood* 115, 2971-2979. 10.1182/blood-2009-05-224824 20042720 PMC2854437

[DMM050361C145] Wang, K.-C., Yeh, Y.-T., Nguyen, P., Limqueco, E., Lopez, J., Thorossian, S., Guan, K.-L., Li, Y.-S. J. and Chien, S. (2016a). Flow-dependent YAP/TAZ activities regulate endothelial phenotypes and atherosclerosis. *Proc. Natl Acad. Sci. USA* 113, 11525-11530. 10.1073/pnas.1613121113 27671657 PMC5068257

[DMM050361C146] Wang, L., Luo, J.-Y., Li, B., Tian, X. Y., Chen, L.-J., Huang, Y., Liu, J., Deng, D., Lau, C. W., Wan, S. et al. (2016b). Integrin-YAP/TAZ-JNK cascade mediates atheroprotective effect of unidirectional shear flow. *Nature* 540, 579-582. 10.1038/nature20602 27926730

[DMM050361C147] Xu, J., Mathur, J., Vessières, E., Hammack, S., Nonomura, K., Favre, J., Grimaud, L., Petrus, M., Francisco, A., Li, J. et al. (2018a). GPR68 Senses flow and is essential for vascular physiology. *Cell* 173, 762-775.e16. 10.1016/j.cell.2018.03.076 29677517 PMC5951615

[DMM050361C148] Xu, S., Xu, Y., Yin, M., Zhang, S., Liu, P., Koroleva, M., Si, S., Little, P. J., Pelisek, J. and Jin, Z. G. (2018b). Flow-dependent epigenetic regulation of IGFBP5 expression by H3K27me3 contributes to endothelial anti-inflammatory effects. *Theranostics* 8, 3007-3021. 10.7150/thno.21966 29896299 PMC5996356

[DMM050361C149] Xu, S., Xu, Y., Liu, P., Zhang, S., Liu, H., Slavin, S., Kumar, S., Koroleva, M., Luo, J., Wu, X. et al. (2019). The novel coronary artery disease risk gene JCAD/KIAA1462 promotes endothelial dysfunction and atherosclerosis. *Eur. Heart J.* 40, 2398-2408. 10.1093/eurheartj/ehz303 31539914 PMC6698662

[DMM050361C158] Zhang, S. H., Reddick, R. L., Piedrahita, J. A. and Maeda, N. (1992). Spontaneous hypercholesterolemia and arterial lesions in mice lacking apolipoprotein E. *Science* 258, 468-471. 10.1126/science.1411543 1411543

[DMM050361C150] Zhang, Y., Zhang, Y., Hutterer, E., Hultin, S., Bergman, O., Kolbeinsdottir, S., Jin, H., Forteza, M. J., Ketelhuth, D. F. J., Roy, J. et al. (2023). The VE-cadherin/AmotL2 mechanosensory pathway suppresses aortic inflammation and the formation of abdominal aortic aneurysms. *Nat. Cardiovasc. Res.* 2, 629-644. 10.1038/s44161-023-00298-8 PMC11358041 39195920

[DMM050361C151] Zhou, J., Li, Y.-S., Wang, K.-C. and Chien, S. (2014). Epigenetic mechanism in regulation of endothelial function by disturbed flow: induction of DNA hypermethylation by DNMT1. *Cell. Mol. Bioeng.* 7, 218-224. 10.1007/s12195-014-0325-z 24883126 PMC4036703

